# Antitumour efficacy of MEK inhibitors in human lung cancer cells and their derivatives with acquired resistance to different tyrosine kinase inhibitors

**DOI:** 10.1038/bjc.2011.244

**Published:** 2011-07-12

**Authors:** F Morgillo, T Cascone, E D'Aiuto, E Martinelli, T Troiani, P Saintigny, R De Palma, J V Heymach, L Berrino, C Tuccillo, F Ciardiello

**Affiliations:** 1 Sezioni di Oncologia Medica, Seconda Università degli Studi di Napoli, Via S. Pansini 5, Naples 80131, Italy; 2 Division of Cancer Medicine, Department of Thoracic/Head and Neck Medical Oncology, The University of Texas MD Anderson Cancer Center, Holcombe Boulevard, Houston, TX 77030, USA; 3 Immunologia Clinica, Seconda Università degli Studi di Napoli, Via S. Pansini 5, Naples 80131, Italy; 4 Sezione di Farmacologia, Dipartimento di Medicina Sperimentale, Seconda Università degli Studi di Napoli, Piazza Miraglia, Naples, Italy; 5 Gastroenterologia, Dipartimento Medico-Chirurgico di Internistica Clinica e Sperimentale ‘F. Magrassi e A. Lanzara’, Seconda Università degli Studi di Napoli, Via S. Pansini 5, Naples 80131, Italy

**Keywords:** acquired resistance, EMT, MEK, erlotinib, gefitinib, vandetanib

## Abstract

**Background:**

To study the molecular mechanisms regulating cancer cell resistance to four different tyrosine kinase inhibitors (TKIs): erlotinib, gefitinib, vandetanib and sorafenib.

**Methods:**

An *in vitro* model of acquired resistance to these TKIs was developed by continuously treating the human lung adenocarcinoma cell line CALU-3 with escalating doses of each drug. Transcriptional profiling was performed with Agilent whole genome microarrays. Western blot analysis, enzyme-linked immunosorbent (ELISA), 3-(4,5-dimethylthiazol-2-yl)-2,5-diphenyltetrazolium bromide (MTT) cell proliferation, migration, invasion and anchorage-independent colony growth assays were conducted *in vitro* and experiments with established xenografts in athymic nude mice were performed *in vivo* in parental (P) and TKI-resistant (R) CALU-3 cell lines.

**Results:**

As compared with P-CALU-3 cells, in TKI-R CALU-3 cell lines a significant increase in the expression of activated, phosphorylated MET, IGF-1R, AKT, MEK, MAPK and of survivin was observed. Downregulation of E-cadherin and amphiregulin mRNAs and upregulation of vimentin, VE-cadherin, HIF-1*α* and vascular endothelial growth factor receptor-1 mRNAs were observed in all four TKI-R CALU-3 cell lines. All four TKI-R CALU-3 cells showed increased invasion, migration and anchorage-independent growth. Together, these data suggest epithelial to mesenchymal transition (EMT) in TKI-R CALU-3 cells. Treatment with several agents that target AKT, MET or IGF-1R did not affect TKI-R CALU-3 cell proliferation. In contrast, treatment with MSC19363669B and selumetinib, two selective MEK inhibitors, caused inhibition of cell proliferation, invasion, migration, anchorage-independent growth *in vitro* and of tumour growth *in vivo* of all four TKI-R CALU-3 cell lines.

**Conclusion:**

These data suggest that resistance to four different TKIs is characterised by EMT, which is MEK-inhibitor sensitive in human CALU-3 lung adenocarcinoma.

Non-small cell lung cancer (NSCLC) is the major cause of cancer-related deaths worldwide (Jemal *et al*, 2009). Platinum-based combination regimens offer a modest but significant survival advantage to NSCLC patients with advanced or metastatic disease although most patients eventually experience disease progression (Stinchcombe *et al*, 2010).

Advances in the understanding of the molecular biology of cancer have enabled the discovery of potential therapeutic molecular targets. Tyrosine kinase inhibitors (TKIs) have been developed to block key intracellular signalling pathways in cancer cells. The epidermal growth factor receptor (EGFR) is involved in the development and the progression of several human cancers, including NSCLC. Two EGFR TKIs, gefitinib (ZD1839, Iressa) and erlotinib (OSI774, Tarceva), represent the first examples of molecularly targeted agents for the treatment of NSCLC and are, currently, used in the management of patients with advanced NSCLC after chemotherapy failure or in the treatment of those patients in which cancer cells harbour specific activating somatic EGFR gene mutations (Ciardiello and Tortora, 2008).

The vascular endothelial growth factor (VEGF) family contributes to tumour-induced neo-angiogenesis, leading to sustained local tumour growth and to metastatic spread of cancer cells (Talmadge and Fidler, 2010). The EGFR and VEGF receptor (VEGFR) share common downstream pathways and exert their effects directly and indirectly on cancer cells (Tonra *et al*, 2006). Several studies have evidenced the role of VEGF in the mechanisms of acquired resistance to EGFR blockade (Bianco *et al*, 2008) and several preclinical studies have been conducted to discover the effects of the combined targeting of the EGFR and VEGF pathways.

Vandetanib (ZD6474, Zactima) is an orally available, dual VEGFR-2 and EGFR TKI. Vandetanib could inhibit tumour cell proliferation and survival by blocking EGFR and could inhibit tumour-induced neo-angiogenesis by blocking VEGF activity and has been investigated in a variety of human cancers, including NSCLC (Wedge *et al*, 2001; Wilhelm *et al*, 2004; Naumov *et al*, 2009; Morabito *et al*, 2010).

Sorafenib (BAY 43-9006, Nexavar) is a multi-targeted kinase inhibitor, which blocks the activation of C-RAF, B-RAF (both the wild-type and the activated V600E mutant), c-KIT, FLT-3, RET, VEGFR-2, VEGFR-3 and PDGFR-β (Wilhelm *et al*, 2004), and is, currently, approved for the treatment of metastatic renal cell carcinoma and for advanced hepatocellular carcinoma. Currently, sorafenib is under investigation in several human malignancies, including NSCLC (Lind *et al*, 2010).

Despite impressive clinical successes with different kinase-targeted therapies, most, if not all, cancer patients with an initially responsive disease eventually experience relapse as a result of acquired drug resistance to these agents (Ciardiello and Tortora, 2008). Consequently, it is critical to define the mechanisms by which drug resistance develops for the identification of therapeutic strategies to avoid and/or to overcome cancer cell acquired resistance.

The aim of this work was to examine the cellular alterations and the molecular mechanisms, which are correlated with acquired resistance induced by four different TKIs, such as erlotinib, gefitinib, vandetanib and sorafenib. For this purpose, we have developed an *in vitro* model of acquired resistance to these TKIs by continuously treating initially responding and sensitive human CALU-3 lung adenocarcinoma cells with escalating doses of each drug.

## Materials and methods

### Cell lines, drugs and chemicals

The human NSCLC CALU-3 cell line was provided by the American Type Culture Collection (Manassas, VA, USA) and maintained in RPMI 1640 supplemented with 10% fetal bovine serum (FBS; Life Technologies, Gaithersburg, MD, USA) in a humidified atmosphere with 5% CO_2_. Gefitinib, vandetanib and selumetinib (AZD6244) were provided by AstraZeneca, Macclesfield, UK; erlotinib was provided by Roche, Basel, Switzerland; sorafenib was provided by Bayer Schering Pharma, Leverkusen, Germany; MSC19363669B (formerly known as AS703026) was provided by EMD Serono, Rockland, MA, USA; deguelin was a generous gift of Dr Ho-Young Lee, University of Texas MD Anderson Cancer Center, Houston, TX, USA; enzastaurin was provided by Lilly Italy, Firenze, Italy; everolimus was provided by Novartis Italy, Milan, Italy; LY294002 was purchased from Calbiochem, END Chemicals Darmstadt, Germany; JNJ-38877605 was purchased from Selleck Chemicals, Houston, TX, USA.

Primary antibodies against P-EGFR (Tyr1173), EGFR, P-MAPK44/42 (Thr202/Tyr204), MAPK44/42, P-AKT (Ser473), AKT, P-MEK (Ser217/221), MEK, P-STAT3 (Tyr705), STAT3, P-IGF1-R (Tyr 1165,1166), IGF1R, P-MET (Tyr1234,1235), MET, HIF-1alpha, VEGFR-1, E-cadherin, caveolin, vimentin, VE-cadherin, survivin were obtained from Cell Signaling Technology, Danvers, MA, USA. Rabbit anti-mouse immunoglobulin G (IgG)–horseradish peroxidase conjugate was provided by DAKO, Carpinteria, CA, USA; donkey anti-rabbit IgG–horseradish peroxidase conjugate and rabbit anti-goat IgG–horseradish peroxidase conjugate were purchased by Amersham Pharmacia Biotech, Arlington Heights, IL, USA. The protein–antibody complexes were detected by enhanced chemiluminescence (ECL kit; Amersham), according to the manufacturer's recommended protocol. Enzyme-linked immunosorbent assay (ELISA) kits for the quantification of amphiregulin, epiregulin, VEGF-A and hepatocyte growth factor (HGF) in the conditioned media, were purchased from R&D Systems, Minneapolis, MN, USA. Cell invasion and migration assay kits were obtained by Chemicon, Millipore, Temecula, CA, USA. APO-bromodeoxyuridine (APO-BrdUrd) staining kit was provided by Phoenix Flow Systems, San Diego, CA, USA. All other chemicals were purchased from Sigma Aldrich, St Louis, MO, USA.

### Establishment of CALU-3 cancer cell lines with acquired resistance to four different TKIs

Over a period of 12 months, human CALU-3 (P-CALU-3) lung adenocarcinoma cells were continuously exposed to increasing concentrations of either gefitinib, erlotinib, vandetanib or sorafenib, as previously described (Morgillo *et al*, 2006). The starting dose was the dose causing the inhibition of 50% of cancer cell growth (IC_50_) for each EGFR inhibitor (i.e., erlotinib, 3 *μ*M; gefitinib, 6 *μ*M; vandetanib, 1 *μ*M; and sorafenib, 0.5 *μ*M). The drug dose was progressively increased to 15 *μ*M in approximately 2 months, to 20 *μ*M after other 2 months, to 25 *μ*M after additional 2 months, and, finally, to 30 *μ*M for a total of 12 months. The established resistant cancer cell lines were then maintained in continuous culture with the maximally achieved dose of each TKI that allowed cellular proliferation (30 *μ*M for each drug).

### Cell proliferation assay

Cancer cells were seeded in 96-well plates and were treated with different drugs, such as erlotinib, gefitinib, vandetanib, sorafenib, enzastaurin, deguelin, everolimus, MSC19363669B or selumetinib for 72 h. Cell proliferation was measured with the 3-(4,5-dimethylthiazol-2-yl)-2,5-diphenyltetrazolium bromide (MTT) assay. The IC_50_ were determined by interpolation from the dose-response curves. Results represent the median of three separate experiments each performed in quadruplicate.

### Western blotting analysis

Following treatment, cancer cells were lysed with Tween-20 lysis buffer (50 mmol l^–1^ HEPES, pH 7.4, 150 mmol l^–1^ NaCl, 0.1% Tween-20, 10% glycerol, 2.5 mmol l^–1^ EGTA, 1 mmol l^–1^ EDTA, 1 mmol l^–1^ DTT, 1 mmol l^–1^ phenylmethylsulfonylfluoride, and 10 *μ*g ml^–1^ of leupeptin and aprotinin) and sonicated. Equal amounts of protein were analysed by SDS–PAGE. Thereafter, proteins were transferred to nitrocellulose membranes and analysed by specific primary antibodies, as indicated in the experiment. Proteins were detected via incubation with horseradish peroxidase-conjugated secondary antibodies and ECL chemiluminescence detection system.

### Microarray analysis of mRNA expression

Agilent microarray analyses (Agilent Technologies, Cernusco, Milan, Italy) were done to assess baseline gene expression profile for each cell line. Briefly, cells were grown to log phase. RNA was extracted using the RNeasy Plus Mini Kit (Qiagen Inc., Milan, Italy) following the manufacturer's instructions. Purity and concentration were determined by Nanodrop1000 spectrophotometer (Thermo Fisher Scientific, Geel, Belgium), while integrity was checked by Agilent Bioanalyzer 2100. A two-colour labelling microarray system was used to compare parental (P) and resistant (R) CALU-3 cell lines. Fluorescently labelled complementary RNA (cRNA) probes were generated by using the Two Color Microarray Quick Labeling kit (KeraFAST, Winston-Salem, NC, USA) and following the manufacturer's instructions. RNA spike-in controls were used to adjust possible dye effects. The spike-in controls represent two sets of ten synthesised RNA mixtures derived from the adenovirus E1A transcriptome with different concentrations in each set (Zahurak *et al*, 2007). These spike-in sets were mixed with either WT- or TKI-R CALU-3 cell lines and co-hybridised to arrays. Briefly, 0.2 *μ*g of total RNA were mixed with spike-ins and converted to cDNA using reverse transcriptase and oligo-dT primers in which T7 promoter sequences were added. T7 RNA polymerase was used for the synthesis and labelling of cRNA with either Cy3 dye for control cell lines or Cy5 dye for the resistant cancer cell lines. The fluorescently labelled cRNA probes were purified using the Qiagen RNeasy Mini Kit (Qiagen Inc.), and the concentration, fluorescent intensities and quality of labelled cRNA probes were determined using a Nanodrop1000 spectrophotometer. An equal amount (825 ng) of Cy3 and Cy5 labelled cRNA probes were hybridised on a 4 × 44 K Agilent human whole genome microarray. The hybridised slides were washed using a commercial kit package (Agilent Technologies) and then scanned using Agilent G2565CA Microarray Scanner System (Agilent Technologies) with the resolution capability of 5 *μ*m. Two biologic replicates were conducted. Data were extracted from slide image using Agilent Feature Extraction software (v.10.5). The gene expression analysis was generated using Array Studio software (Omicsoft Corporation, Research Triangle Park, NC, USA). Lowess normalisation was used to compensate for non-linear dye-bias. For each drug, two technical replicates were combined to give a unique red and green mean estimate of gene expression, and the log_2_ fold-change between resistant and sensitive cell lines was computed. Probes with an absolute log_2_ fold-change equal or superior to 0.5 were included in subsequent analyses. Venn diagrams were generated to study the overlap between genes and probes up- or downregulated in cells resistant to erlotinib, gefitinib, vandetanib or sorafenib. Statistical significance was set at a *P*<0.001 value.

### Evaluation of growth factor secretion

The concentrations of amphiregulin, epiregulin, VEGF-A and HGF in the conditioned medium were measured using available commercially ELISA kits according to the manufacturer's instructions. In all, 3 × 10^5^ cells were plated in six-well plates in complete medium (10% RPMI), which was changed to serum-free medium before the assessment of growth factor production. Conditioned medium was then harvested, concentrated and tested by ELISA for quantification. Assays were performed in triplicate. Results were normalised for the number of producing cells and reported as pg of ligands per 10^6^ cells per 72 h.

### Invasion assay

The invasive ability *in vitro* was measured by using transwell chambers, according to the manufacturer's protocol. Briefly, cells were seeded onto the membrane of the upper chamber of the transwell at a concentration of 2 × 10^5^ per ml in 500 *μ*l of RPMI medium and were left untreated or treated with the indicated doses of MSC19363669B or selumetinib for 24 h. The medium in the upper chamber was serum-free. The medium at the lower chamber contained 10% FBS as a source of chemo-attractants. After 24 h, cells that passed through the Matrigel-coated membrane were stained with Cell Stain Solution containing crystal violet supplied in the transwell invasion assay (Chemicon, Millipore) and photographed. Absorbance was measured at 562 nM by an ELISA reader after dissolving of stained cells in 10% acetic acid. Assays were performed in triplicate.

### Migration assay

Cell migration was assessed using a commercially available chemotaxis assay. Briefly, cells were incubated in RPMI serum-free medium for 24 h and were left untreated or treated with the indicated doses of MSC19363669B or selumetinib, following which they were detached from flasks, suspended in quenching medium (serum-free medium containing 5% bovine serum albumin) and EDTA, and seeded into Boyden migration chamber inserts placed in a 24-well plate. The inserts contain a microporous membrane with an 8-*ì*m pore size. Inserts were placed over wells containing serum-free media plus chemo-attractant (10% FBS). After a 48-h treatment period, cells per media were discarded from the top side of the migration chamber insert and the chamber was placed in the wells of a new 24-well plate containing cell detachment solution. Following incubation for 30 min at 37 °C, the insert was discarded, and a solution of lysis buffer and CyQuant GR dye was added to each well. CyQuant is a green fluorescent dye that exhibits strong enhancement of fluorescence when bound to cellular nucleic acids released by the lysis buffer, enabling assessment of the relative number of migrated cells. Fluorescence was determined with a fluorimeter at 480/520 nM. Assays were performed in triplicate.

### Growth in soft agar

Cells (10^4^ cells per well) were suspended in 0.5 ml 0.3% Difco Noble agar (Difco, Detroit, MI, USA) supplemented with complete culture medium. This suspension was layered over 0.5 ml 0.8% agar-medium base layer in 24-multiwell cluster dishes (Becton Dickinson, Lincoln Park, NJ, USA) and treated with different concentrations of MSC19363669B or selumetinib. After 14 days, cells were stained with nitro blue tetrazolium (Sigma) and colonies larger than 0.05 mM were counted. Assays were performed in triplicate.

### Apoptosis assay

Both adherent and nonadherent cells were harvested, pooled, and fixed with 1% paraformaldehyde and 70% ethanol. Apoptosis was assessed with a flow cytometry-based terminal deoxyribonucleotide transferase-mediated nick-end labelling (TUNEL) assay processed with an APO-BrdUrd staining kit (Phoenix Flow Systems). Cells treated with DMSO were used as a negative control, and for a positive control, we used the HL-60 leukaemic cells treated with camptothecin provided with the kit.

### Tumour xenografts in nude mice

Four- to six-week old female balb/c athymic (nu+/nu+) mice were purchased from Charles River Laboratories (Milan, Italy). The research protocol was approved and mice were maintained in accordance with the institutional guidelines of the Second University of Naples Animal Care and Use Committee. Mice were acclimatised for 1 week before being injected with cancer cells and injected subcutaneously with 10^7^ CALU-3 (P, ERL-R, GEF-R, VAN-R or SOR-R) cells that had been resuspended in 200 *μ*l of Matrigel (Becton Dickinson). When established tumours of approximately 75 mm^3^ in diameter were detected, mice were treated with oral administrations of MSC19363669B (15 mg kg^–1^ per bid, days 1 to 5 of each week), for the indicated time periods. Each treatment group consisted of eight mice. Tumour volume was measured using the formula π/6 × larger diameter × (smaller diameter)^2^.

### Statistical analysis

The Student's *t*-test was used to evaluate the statistical significance of the results. All *P*-values represent two-sided tests of statistical significance. All analyses were performed with the BMDP New System statistical package version 1.0 for Microsoft Windows (BMDP Statistical Software, Los Angeles, CA, USA).

## Results

### Development and characterisation of TKI-R CALU-3 cancer cells

The human NSCLC cell line CALU-3 harbours the wild-type EGFR gene and an activating K-RAS (p.G13D) gene mutation. This cancer cell line has been previously characterised by our group for the expression of the four EGF-related growth factor receptors (EGFR, ERBB2, ERBB3 and ERBB4) and of three VEGF receptors (VEGFR-1, VEGFR-2 and VEGFR-3), as well as for the expression of three EGFR ligands (amphiregulin, EGF and TGF*α*) and of three VEGFR ligands (VEGF-A, VEGF-B and VEGF-C), by using quantitative RT–PCR (Martinelli *et al*, 2010). All tested ligand mRNAs, with the exception of VEGF-C, were expressed in CALU-3 cells (Supplementary Figure 1). CALU-3 cells also expressed EGFR mRNA; whereas low levels of ERBB2 and ERBB3 mRNAs were measurable. No detectable expression of ERBB4 mRNA was found. Moreover, VEGFR-1 and VEGFR-2 mRNA expression was detected. Expression of EGFR and its specific ligands suggests that in human lung adenocarcinoma CALU-3 cells an EGFR-driven autocrine pathway is relevant for cancer cell proliferation. In fact, CALU-3 cells are growth inhibited by treatment with selective EGFR TKIs, such as gefitinib or erlotinib (Martinelli *et al*, 2010; Morgillo *et al*, 2010). Furthermore, CALU-3 cancer cells express both VEGF ligands and VEGFRs and are growth inhibited by treatment with anti-angiogenic TKIs (Martinelli *et al*, 2010).

**Figure 1:**
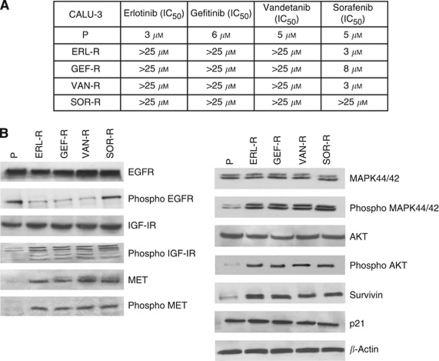
Development and characterisation of TKI-R CALU-3 cancer cells. (**A**) Inhibitory concentration 50 values (IC_50_) for treatment with erlotinib, gefitinib, vandetanib or sorafenib in parental human lung adenocarcinoma CALU-3 cells (P) and in their TKI-R derivatives (ERL-R, GEF-R, VAN-R and SOR-R). Cell proliferation was measured with the MTT assay. The drug concentrations required to inhibit cell growth by 50% (IC_50_) were determined by interpolation from the dose-response curves. Results represent the average of three separate experiments each performed in quadruplicate. (**B**) Western blotting analysis of selected growth factor receptors (EGFR, IGF-1R and MET) and of down-stream signalling pathways in parental human lung adenocarcinoma CALU-3 cells (P) and in their TKI-R derivatives (ERL-R, GEF-R, VAN-R and SOR-R). *β*-Actin was included as a loading control.

Therefore, CALU-3 cells were selected as a model for exploring the acquired resistance mechanisms to treatment with the EGFR TKIs erlotinib and gefitinib, or with the dual EGFR/VEGFR TKI vandetanib, or with the multi-kinase inhibitor sorafenib. The gefitinib- (GEF-R), erlotinib- (ERL-R), vadetanib- (VAN-R) and sorafenib- (SOR-R) resistant cell lines were obtained by continuously culturing CALU-3 cells in the presence of increasing doses of each drug for 12 months. After the establishment of four different TKI-R CALU-3 cell lines, we characterised their resistant phenotype by doing cell proliferation assays in the presence of each of these inhibitors. As illustrated in Figure 1A, an approximately 10-fold increase in the IC_50_ for each TKI-R CALU-3 cell line as compared with parental CALU-3 cells was observed. ERL-R, GEF-R and VAN-R CALU-3 human cancer cell lines were cross-resistant to either gefitinib, erlotinib or vandetanib treatment. In contrast, sorafenib treatment was able to inhibit cell proliferation of ERL-R, GEF-R and VAN-R CALU-3 cell lines. Moreover, SOR-R CALU-3 cells were also resistant to gefitinib, erlotinib or vandetanib treatment. We next confirmed the establishment of stable TKI-R CALU-3 cancer cells in a drug-free culture medium. In fact, all four TKI-R CALU-3 cell lines could grow in the absence of each drug for long periods of time (3 to 6 months) and maintain their TKI-R phenotype (data not shown).

To further characterise the TKI-R CALU-3 cell lines, we examined differential protein expression among parental, sensitive CALU-3 cells and their TKI-R derivatives. Activation of the EGFR leads to a complex intracellular signalling, which includes the activation of the pro-survival PI3K/AKT pathway and the mitogenic RAS/RAF/MEK/MAPK pathway (Janmaat *et al*, 2006; Gandhi *et al*, 2009). We, therefore, investigated by immunoblotting analysis these molecular pathways. As illustrated in Figure 1B, EGF-stimulated activation of the EGFR was efficiently blocked in P- and ERL-R, GEF-R and VAN-R CALU-3 cells, but not in SOR-R CALU-3 cells, as demonstrated by the inhibition of EGFR auto-phosphorylation (P-EGFR). It has been suggested that increased expression and/or activation of other cell membrane growth factor receptors, such as insulin-like growth factor-1 receptor (IGF-1R) and/or MET, could be responsible for the acquired resistance to EGFR-targeted therapies (Morgillo *et al* 2007; Engelman *et al*, 2007; Engelman and Jänne, 2008). IGF-1R and MET result activated in all four TKI-R CALU-3 cell lines with increased level of both phosphorylated IGF-1R (P-IGF-1R) and MET (P-MET). Of interest, all four TKI-R CALU-3 cell lines showed also an increased expression of MET protein.

As the activated, phosphorylated forms of AKT and MAPK are key intracellular mediators of growth factor-activated cell survival and proliferation signals (Janmaat *et al*, 2006), investigating the activation state of these molecular pathways may be of interest in the understanding the resistance mechanisms. Activation of MAPK and AKT, indicated by an increase in P-MAPK and P-AKT, as well as an increase in survivin protein levels were observed in all four TKI-R CALU-3 cell lines as compared with their parental counterpart (Figure 1B). Taken together, these results suggest that in this cancer cell model of acquired resistance to four different TKIs, activation of AKT- and MAPK-driven intracellular signals, which could be activated also by other cell membrane growth factor receptors such as IGF-1R and/or MET, may be responsible for cancer cell growth in the presence of either selective anti-EGFR TKIs, such as gefitinib or erlotinib, or in the presence of broad spectrum TKIs, such as vandetanib or sorafenib.

### Identification of differential gene expression in TKI-R CALU-3 cancer cells

Further experiments were conducted to better explore the possible mechanisms of acquired resistance. Basal mRNA gene expression profiles were obtained from P-CALU-3 cells and their four TKI-R CALU-3 derivatives by using Agilent microarrays. A first analysis was performed to define genes, which had a significantly (*P*<0.001) different mRNA expression in ERL-R and GEF-R CALU-3 cell lines as compared with P-CALU-3 cells. As shown in Supplementary Figure 2, on a total list of 43 376 genes analysed, in GEF-R and ERL-R CALU-3 cells the mRNA expression of 539 and 390 genes was upregulated as compared with P-CALU-3 cells, respectively; whereas the mRNA expression of 673 and 1047 genes was downregulated in GEF-R and ERL-R CALU-3 cells, respectively. Among these genes, the two EGFR inhibitor-R CALU-3 cell lines shared a panel of 194 upregulated genes and of 326 downregulated genes, respectively (Supplementary Figure 2). A list of the most significant upregulated and downregulated mRNAs is shown in Supplementary Tables 1A and B. Among the genes whose mRNA expression was upregulated in GEF-R and ERL-R CALU-3, there were vimentin, caveolin, HIF-1α and VEGF-B, which are generally correlated to a more invasive cancer cell phenotype. In line with these results and with an epithelial to mesenchymal transition (EMT), E-cadherin, amphiregulin and epiregulin mRNA expression was downregulated.

We next performed a similar analysis for evaluating gene expression differences between VAN-R and SOR-R CALU-3 cell lines as compared with P-CALU-3 cells. In VAN-R and SOR-R CALU-3 cell lines, the mRNA expression of 653 and 363 genes, was significantly (*P*<0.001) upregulated, respectively; whereas the mRNA expression of 1072 and 558 genes, was significantly (*P*<0.001) downregulated, respectively (Supplementary Figure 3). We identified 135 upregulated genes and 298 downregulated genes, which were overlapping between these two TKI-R CALU-3 cell lines (Supplementary Figure 3). A list of the most significant upregulated and downregulated mRNAs is shown in Supplementary Tables 2A and B. Similarly to ERL-R and GEF-R CALU-3 cell lines, VE-cadherin, vimentin, caveolin and HIF-1α mRNA expression was upregulated in VAN-R and SOR-R CALU-3 cell lines. These results suggest a shift to EMT also in CALU-3 cells with acquired resistance to the multi-targeted anti-angiogenic TKIs vandetanib and sorafenib. In this respect, similarly to ERL-R and GEF-R CALU-3 cell lines, downregulation of amphiregulin mRNA expression was observed also in VAN-R and SOR-R CALU-3 cell lines.

Collectively, the CALU-3-derived cancer cell lines with acquired resistance to four different TKIs shared 133 upregulated and 72 downregulated mRNAs as compared with P-CALU-3 cell gene expression (Supplementary Figure 4). Among the downregulated mRNAs, there were epithelial-related genes, such as E-cadherin and amphiregulin (Supplementary Table 3A). Conversely, among the upregulated genes there were mesenchymal-related genes, including vimentin, caveolin, VE-cadherin, and angiogenesis-related genes, such as VEGFR-1 and HIF-1α (Supplementary Table 3B). Taken together, these data show the loss of epithelial features and the acquisition of a mesenchymal behaviour, which are consistent with EMT phenotype in all four TKI-R CALU-3 cancer cell lines.

### Expression of EMT markers in TKI-R CALU-3 cancer cells

**Figure 2:**
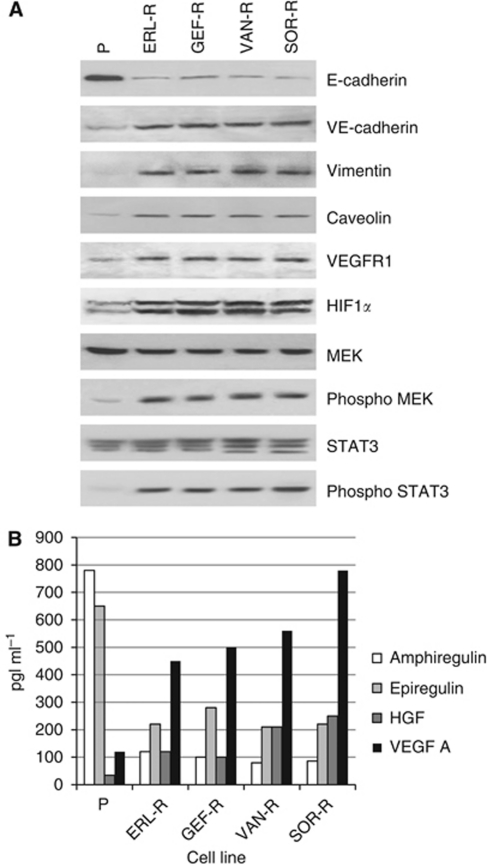
Differential expression of proteins in parental and TKI-R CALU-3 cancer cells. (**A**) Western blotting analysis of the expression of selected epithelial- and mesenchymal-related proteins in parental human lung adenocarcinoma CALU-3 cells (P) and in their TKI-R derivatives (ERL-R, GEF-R, VAN-R and SOR-R). (**B**) Secretion of amphiregulin, epiregulin, HGF and VEGF-A into the conditioned medium, as measured in cell culture media by specific ELISAs.

To further evaluate the potential role of EMT in causing TKI resistance, the expression of epithelial- and mesenchymal-related proteins was assessed in P-CALU-3 cells and in their four TKI-R-derived cell lines. As shown in Figure 2A, expression of the epithelial-related protein E-cadherin was lost in all four TKI-R CALU-3 cell lines. Conversely, the expression of the mesenchymal markers vimentin and caveolin and of the angiogenesis markers VE-cadherin, HIF-1α and VEGFR-1 was detected in all four TKI-R CALU-3 cell lines with no evidence in P- CALU-3 cells. Furthermore, in all four TKI-R CALU-3 cell lines a significant increase in activated, phosphorylated MEK and in its down-stream effector STAT3 was observed as compared with P-CALU-3 cells.

We next analysed the secretion into the cell culture media of two EGFR selective ligands, amphiregulin and epiregulin, of the major VEGFR-2 ligand, VEGF-A and of HGF, the specific ligand for the MET growth factor receptor, which is involved in the control of cell growth and motility. As shown in Figure 2B, both amphiregulin and epiregulin were secreted by P-CALU-3 cells, but their production was significantly decreased in all four TKI-R CALU-3 cell lines. On the contrary, VEGF-A and HGF secretion was significantly increased in all four TKI-R CALU-3 cell lines as compared with P-CALU-3 lung adenocarcinoma cells.

### Differential invasive, migratory and anchorage-independent growth properties in TKI-R CALU-3 cancer cells

**Figure 3:**
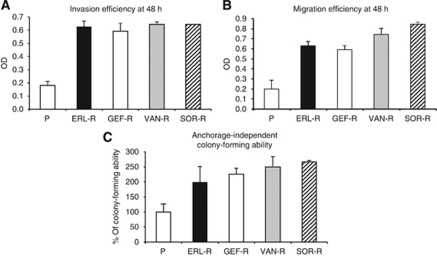
Differential invasive and migratory properties, and anchorage-independent growth as colonies of parental and TKI-R CALU-3 cancer cells. (**A**) Invasion assay; (**B**) migration assay; (**C**) anchorage-independent colony formation in soft-agar, in parental human lung adenocarcinoma CALU-3 cells (P) and in their TKI-R derivatives (ERL-R, GEF-R, VAN-R and SOR-R). The results are the average±s.d. of three independent experiments, each done in triplicate.

It has been suggested that cancer cells undergoing EMT could gain a more aggressive and metastatic phenotype with increased ability to invade, migrate and to form colonies in semisolid medium (Polyak and Weinberg, 2009). Therefore, we evaluated these properties in TKI-sensitive P-CALU-3 cancer cells and in their TKI-R derivatives. As illustrated in Figures 3A and B, P-CALU-3 cells demonstrated little or no ability in invasion and migration. On the contrary, all four TKI-R CALU-3 cell lines exhibited significant invasive and migratory abilities. Moreover, their anchorage-independent colony growth was increased of approximately three-fold as compared with P-CALU-3 cells (Figure 3C). Collectively, these results suggest that CALU-3 lung adenocarcinoma cells with acquired resistance to erlotinib, gefitinib, vandetanib or sorafenib have lost epithelial differentiation and have acquired mesenchymal properties, which enable these cells to a more invasive and, potentially, more metastatic behaviour.

### Effects of MEK inhibition on TKI-R CALU-3 cancer cell growth

**Figure 4:**
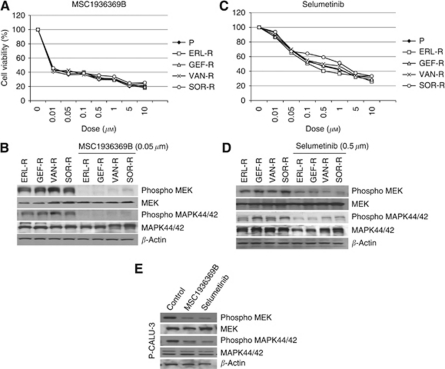
Growth inhibitory effects of treatment with selective MEK inhibitors in parental and TKI-R CALU-3 cancer cells. MTT cell proliferation assays were performed in parental lung adenocarcinoma CALU-3 cells (P) and in their TKI-R derivatives (ERL-R, GEF-R, VAN-R and SOR-R), treated for 3 days with the indicated concentrations of each of two selective MEK inhibitors, MSC19363669B (**A**) or selumetinib (**C**). Results represent the average (±s.d.) of three separate experiments, each performed in quadruplicate. Western blotting analysis of MEK and MAPK activation following treatment with the indicated concentration of each of two selective MEK inhibitors, MSC19363669B (**B**) or selumetinib (**D**) on TKI-R CALU-3 and on parental cell line (**E**). *β*-Actin was included as a loading control.

To determine whether CALU-3 cancer cells could be sensitive to drugs that selectively inhibit the IGF-1R or MET receptor, or to drugs that target the intracellular signalling pathways, which resulted activated following the acquisition of a TKI-R phenotype, we tested the anti-proliferative effects of several agents, including AG1024, an inhibitor of IGF-1R (Morgillo *et al*, 2006), JNJ-38877605, an inhibitor of MET (Comoglio *et al*, 2008) (Supplementary Figure 5), everolimus, an inhibitor of mTOR (Smolewski, 2006), deguelin, an AKT inhibitor (Lee *et al*, 2005), MSC19363669B and selumetinib, two MEK inhibitors (Yeh *et al*, 2007; Kim *et al*, 2010) on the parental P- and TKI-R CALU-3 cancer cells. Different degrees of cell growth inhibition were observed. Although cell proliferation of ERL-R, GEF-R, VAN-R and SOR-R CALU-3 cell lines was little or no affected by AG1024, JNJ-38877605, everolimus or deguelin single-agent treatment (Supplementary Figure 5, for AG1024 and JNJ-3877605), a significant inhibition of cell growth was observed in both P-CALU-3 cells and their four TKI-R derivatives following MSC19363669B or selumetinib treatment, with an IC_50_ ranging from 0.01 to 0.1 *μ*M, (Figures 4A and C). We further characterized the effects of MEK inhibitor treatment on intracellular signalling by Western blotting. As illustrated in Figures 4B, D and E, treatment of, ERL-R, GEF-R, VAN-R, SOR-R and P- CALU-3 cells with MSC19363669B or with selumetinib for 48 h did not affect total MEK and MAPK protein levels, while it caused a marked decrease in the phosphorylated, activated forms of MEK (P-MEK) and of MAPK (P-MAPK).

### Effects of MEK inhibition on the invasion, migration and anchorage-independent growth of TKI-R CALU-3 cancer cells

**Figure 5:**
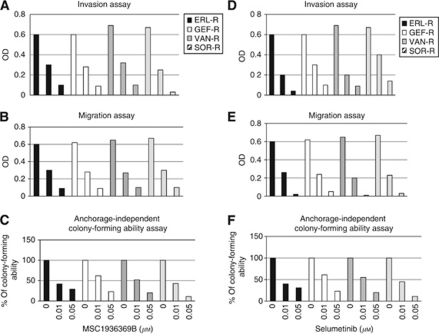
Effects of treatment with selective MEK inhibitors on the invasive, migratory and anchorage-independent colony-forming capabilities of TKI-R CALU-3 cancer cells. Invasion (**A** and **D**), migration (**B** and **E**) and anchorage-independent growth (**C** and **F**) were evaluated in TKI-R lung adenocarcinoma CALU-3 derivatives (ERL-R, GEF-R, VAN-R and SOR-R) after treatment with the indicated concentrations of MSC19363669B (**A**–**C**) or selumetinib (**D**–**F**). The results are the average±s.d. of three independent experiments, each done in triplicate.

We next evaluated the effects of MEK inhibition on the invasive and migratory capabilities of the four TKI-R CALU-3 cell lines. A significant dose-dependent inhibition of invasion and migration was observed in all four TKI-R CALU-3 cell lines following treatment with either MSC19363669B or selumetinib (Figures 5A, B, D and E). Similarly, treatment with MSC19363669B or with selumetinib inhibited the anchorage-dependent growth as colonies in soft-agar of ERL-R, GEF-R, VAN-R and SOR-R CALU-3 cells (Figures 5C and F).

### Effects of MEK inhibition on the apoptosis of TKI-R CALU-3 cancer cells

**Figure 6:**
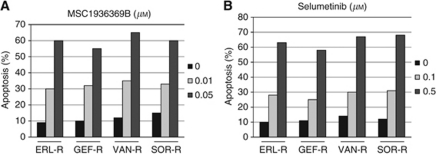
Pro-apoptotic activity of two MEK inhibitors, MSC19363669B (**A**) and selumetinib (**B**), on TKI-R CALU-3. TKI-R CALU-3 cells were treated for the indicated time with two MEK inhibitors, MSC19363669B and selumetinib, at the showed doses. Apoptosis was assessed by a modified TUNEL assay as described in Materials and Methods section.

We next evaluated the effects of MEK inhibition on the apoptosis of the four TKI-R CALU-3 cell lines. Terminal deoxyribonucleotide transferase-mediated nick-end labelling staining and flow cytometric analysis revealed that from 20 to 30% of TKI-R CALU-3 cancer cells underwent apoptosis after treatment with 0.01 *μ*M of MSC19363669B or with 0.1 of selumetinib. Treatment with 0.05 *μ*M of MSC19363669B and with 0.5 *μ*M of selumetinib increased the percentage of apoptotic cells respectively to 60% and 70% (Figure 6).

### Effects of MEK inhibition on TKI-R CALU-3 tumour xenografts

**Figure 7:**
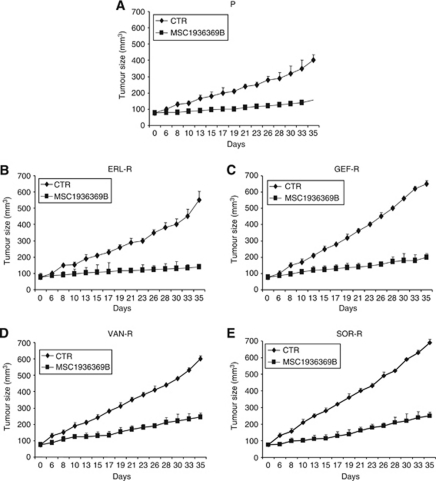
Antitumour activity of the selective MEK inhibitor MSC19363669B in parental and TKI-R CALU-3 xenografts. (**A**) Parental (P) CALU-3 cancer cells; (**B**) ERL-R CALU-3 cancer cells; (**C**) GEF-R CALU-3 cancer cells; (**D**) VAN-R CALU-3 cancer cells; (**E**) SOR-R CALU-3 cancer cells. Athymic nude mice were injected subcutaneously into the dorsal flank with 10^7^ cancer cells. After 7 to 10 days (average tumour size, 75 mm^3^), mice were treated as indicated in Materials and Methods section for 5 weeks. Each treatment group consisted of eight mice. Data represent the average (±s.d.). Student's *t*-test was used to compare tumour sizes among different treatment groups at day 35 following the start of treatment. (**A**) P-CALU-3: MSC19363669B *vs* control (two-sided *P*<0.001); (**B**) CALU-3 ERL-R: MSC19363669B *vs* control (two-sided *P*<0.001). (**C**) CALU-3 GEF-R: MSC19363669B *vs* control (two-sided *P*<0.001); (**C**) CALU-3 VAN-R: MSC19363669B *vs* control (two-sided *P*<0.001); (**D**) CALU-3 SOR-R: MSC19363669B *vs* control (two-sided *P*<0.001).

We finally investigated the *in vivo* antitumour activity of MSC19363669B in nude mice bearing P-CALU-3 or TKI-R CALU-3 cell lines, which were grown subcutaneously as xenografts. In P-CALU-3 tumour xenografts, treatment with MSC19363669B caused a significant decrease in tumour size as compared with control untreated mice. For example, at day 35 from the starting of treatment, the mean tumour volume in mice bearing P-CALU-3 tumour xenografts and treated with MSC19363669B was 38% as compared with control untreated mice (Figure 7A). Also in mice bearing ERL-R, GEF-R, VAN-R or SOR-R CALU-3 tumour xenografts, treatment with MSC19363669B induced a significant reduction in tumour growth (Figures 7B–E). In this respect, at day 35 from the starting of treatment, the mean tumour volumes in the MSC19363669B-treated mice ranged between 27 and 40%, as compared with control untreated mice.

## Discussion

Although paracrine and autocrine activation of the EGFR and the VEGFR pathways have a key role in the development and progression of the majority of epithelial cancers including NSCLC, only a subgroup of NSCLC patients benefits of treatments with drugs targeting the EGFR or the VEGFR pathways (Morgillo *et al*, 2007). Even in initially responding NSCLC patients secondary or acquired resistance occurs causing cancer progression and treatment failure. Several molecular mechanisms have been suggested to contribute to the acquisition of cancer cell resistance to molecularly targeted anticancer drugs (Morgillo *et al*, 2007).

To our knowledge, this is the first work documenting the development of human cancer cells resistant to different TKIs from the same parental cell line, and in particular studying the resistance to the multi-kinase inhibitors, sorafenib. In this study, we have found that a common phenotype, which is correlated to acquired resistance following long term *in vitro* treatment with four different TKIs, such as two EGFR inhibitors (erlotinib and gefitinib) and two broad spectrum kinase inhibitors (vandetanib and sorafenib), of human CALU-3 lung adenocarcinoma cells, is represented by the acquisition of EMT cellular properties with the combined loss of epithelial cell junction proteins, such as E-cadherin, and the gain of mesenchymal markers, such as vimentin. These findings are in agreement with and extend the results from other cancer cell models, including pancreatic (Yin *et al*, 2007), colorectal (Buck *et al*, 2007a, 2007b), head and neck (Frederick *et al*, 2007), bladder (Shrader *et al*, 2007) and breast (Buck *et al*, 2007a, 2007b) cancers, in which expression of EMT markers correlated with lack of activity of EGFR targeting drugs. A potential clinical validation for this experimental hypothesis resulted from a retrospective analysis of TRIBUTE, a randomised phase III trial, which compared the combination of erlotinib plus platinum-based doublet chemotherapy to chemotherapy alone as first-line treatment of metastatic NSCLC patients (Herbst *et al*, 2005). This trial failed to show a significant clinical benefit for the concurrent administration of erlotinib plus chemotherapy in the unselected NSCLC patient population. However, NSCLC patients, whose tumour samples had strong E-cadherin cancer cell staining by immunohistochemistry, had a significantly longer time to progression and a trend to increased overall survival, when treated with the combination of erlotinib plus chemotherapy as compared with chemotherapy alone (Yauch *et al*, 2005).

A second finding from this study is the increase in phosphorylated, activated IGF-1R and MET cell membrane growth factor receptors in CALU-3 cancer cells with acquired resistance to the four TKIs. Interestingly, IGF-1R could promote EMT, although committed mesenchymal-like lung, colon and pancreatic adenocarcinoma cells are no longer dependent on IGF-1R signalling for proliferation or survival (Barr *et al*, 2008), suggesting that, like EGFR, IGF-1R signalling is an important driver in epithelial cells and can induce EMT, but once these cells have transitioned to a mesenchymal state they are no longer reliant on IGF-1R, whereas alternate growth factor pathways could be activated following EMT. In this respect, recent data suggest that PDGFR can exert proliferative and anti-apoptotic effects (Matei *et al*, 2006; Thomson *et al*, 2008). This mechanism may in part explain the efficacy of sorafenib in inhibiting proliferation of ERL-R, GEF-R and VAN-R CALU-3 cell lines.

In line with EMT, all four TKI-R CALU-3 cell lines exhibited a decreased secretion of amphiregulin and epiregulin together with an increased secretion of VEGF-A and HGF. High amphiregulin expression has been suggested as a positive predictor of sensitivity to anti-EGFR drugs in NSCLC patients, whose tumour had the P-EGFR gene (Yonesaka *et al*, 2008). Similarly, several studies have reported an increased efficacy of cetuximab treatment of CRC patients, whose tumour had the WT K-RAS gene and expressed high levels of amphiregulin and/or epiregulin (Jacobs *et al*, 2009). In this respect, human cancer cells, which exhibit mesenchymal-like characteristics, express lower levels of EGF ligands, such as amphiregulin and epiregulin (Oliveras-Ferraros *et al*, 2011). These results suggest that these cells are dependent upon alternate cell membrane growth factor signalling pathways rather than from the EGFR-driven pathway.

Further, all four TKI-R CALU-3 cell lines exhibited elevated levels of phosphorylated, activated AKT, MEK and MAPK proteins, suggesting that the constitutive activation of these key intracellular signals could be an important mechanism in the acquisition of resistance to these TKIs. It has been reported that epithelial cancer cells, which have a transition to a mesenchymal-like phenotype, could have the upregulation of the PI3K/AKT cell survival pathway (Ono *et al*, 2004). However, single-agent inhibition of the PI3K/AKT pathway in TKI-R CALU-3 cells did not cause a significant inhibition in cell proliferation, suggesting that this pathway is not the main responsible for cancer cell survival in this experimental cancer cell system.

On the contrary, inhibition of the MEK/MAPK pathway by treatment with selective MEK inhibitors significantly blocked cell proliferation in all four TKI-R CALU-3 cell lines. Moreover, treatment with selective MEK inhibitors significantly decreased invasion, migration and formation of colonies in semi-solid medium of all four TKI-R CALU-3 cell lines. Moreover, selective MEK inhibition was also able to significantly block TKI-R CALU-3 cell growth as tumour xenografts *in vivo*, indicating a key role of the MEK/MAPK pathway in the EMT in this experimental cancer cell system.

In summary, this study suggests that in an epithelial cancer cell model a common mechanism of acquired resistance to the growth inhibitory effects of four TKIs with different target activity spectrum, including the EGFR and VEGFR, is represented by a MEK-dependent transition from an epithelial to a mesenchymal phenotype. These results may have potential clinical relevance. Treatment with selective MEK inhibitors has a significant antitumour activity *in vitro* and *in vivo*. Therefore, the use of selective MEK inhibitors could be a potentially effective therapeutic strategy for preventing and/or overcoming cancer resistance to different TKIs.

## Supplementary Material

Supplementary Figure 5

Supplementary Table 1A

Supplementary Table 1B

Supplementary Table 2A

Supplementary Table 2B

Supplementary Table 3A

Supplementary Table 3B
