# Supplementary material for: Antitumour efficacy of MEK inhibitors in human lung cancer cells and their derivatives with acquired resistance to different tyrosine kinase inhibitors
Source: Br J Cancer. 2011 Jul 12;105(3):382–92. doi: 10.1038/bjc.2011.244 (PMC3172903; doi:10.1038/bjc.2011.244)
Supplement: Supplementary Figure 1 [file bjc2011244x1.ppt]

## Slide 1
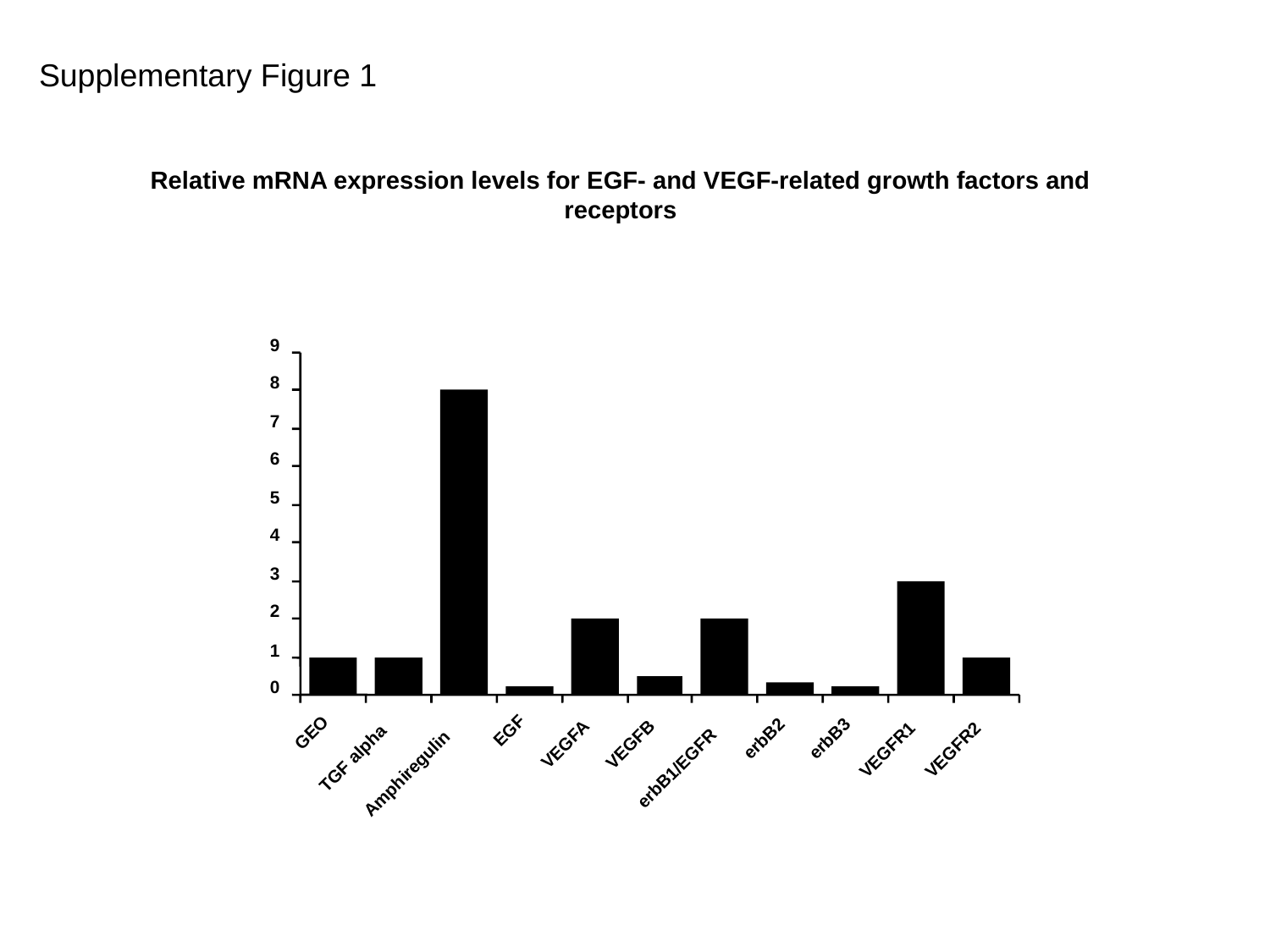

Supplementary Figure 1
Relative mRNA expression levels for EGF- and VEGF-related growth factors and receptors
9
8
7
6
5
4
3
2
1
0
EGF
GEO
erbB2
erbB3
VEGFA
VEGFB
VEGFR1
VEGFR2
TGF alpha
erbB1/EGFR
Amphiregulin
