# Supplementary material for: Antitumour efficacy of MEK inhibitors in human lung cancer cells and their derivatives with acquired resistance to different tyrosine kinase inhibitors
Source: Br J Cancer. 2011 Jul 12;105(3):382–92. doi: 10.1038/bjc.2011.244 (PMC3172903; doi:10.1038/bjc.2011.244)
Supplement: Supplementary Figure 2 [file bjc2011244x2.ppt]

## Slide 1
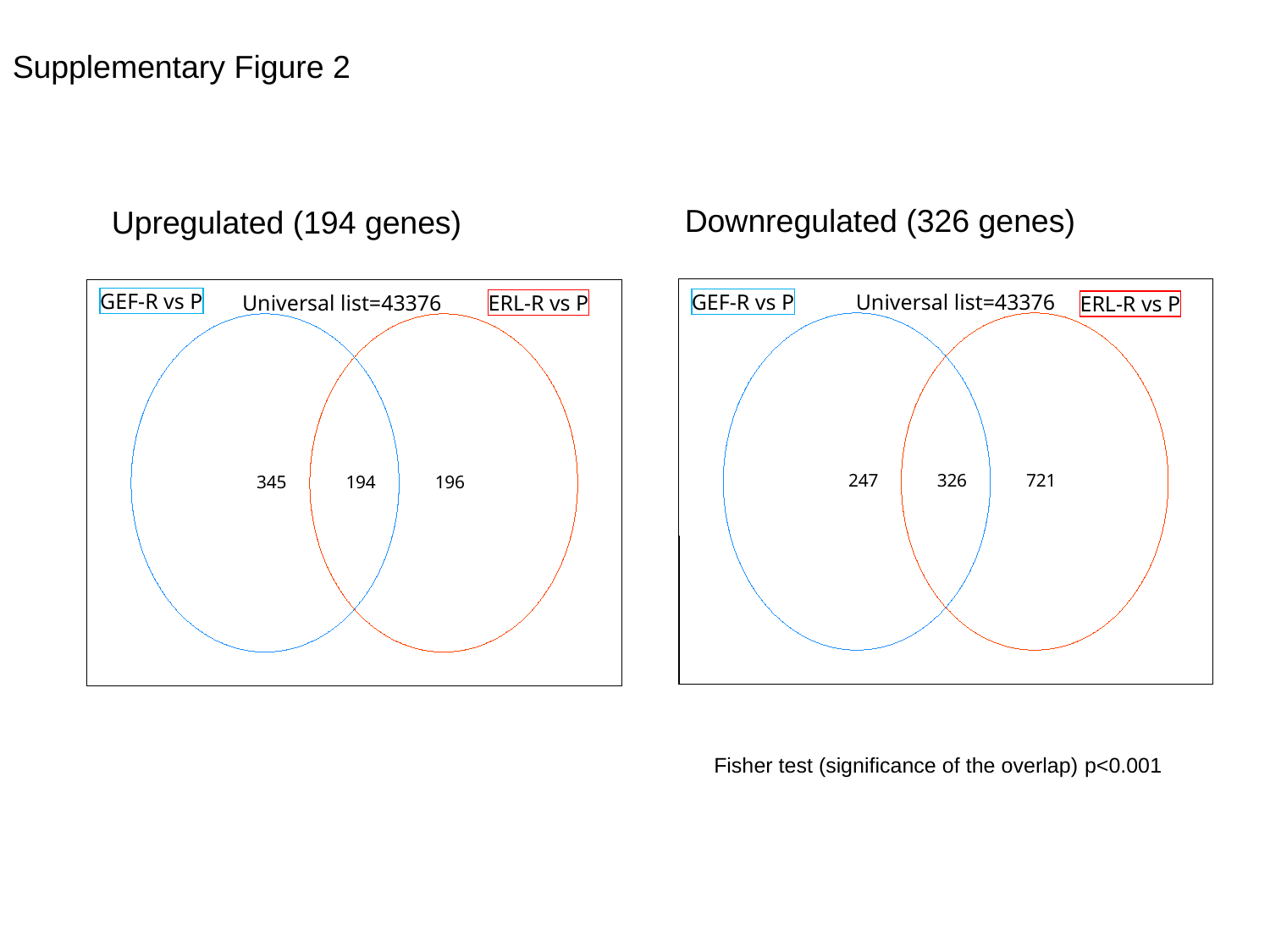

# Supplementary Figure 2
Downregulated (326 genes)
Upregulated (194 genes)
GEF-R vs P
Universal list=43376
GEF-R vs P
Universal list=43376
ERL-R vs P
ERL-R vs P
247
326
721
345
194
196
Fisher test (significance of the overlap) p<0.001
