# Supplementary material for: Antitumour efficacy of MEK inhibitors in human lung cancer cells and their derivatives with acquired resistance to different tyrosine kinase inhibitors
Source: Br J Cancer. 2011 Jul 12;105(3):382–92. doi: 10.1038/bjc.2011.244 (PMC3172903; doi:10.1038/bjc.2011.244)
Supplement: Supplementary Figure 3 [file bjc2011244x3.ppt]

## Slide 1
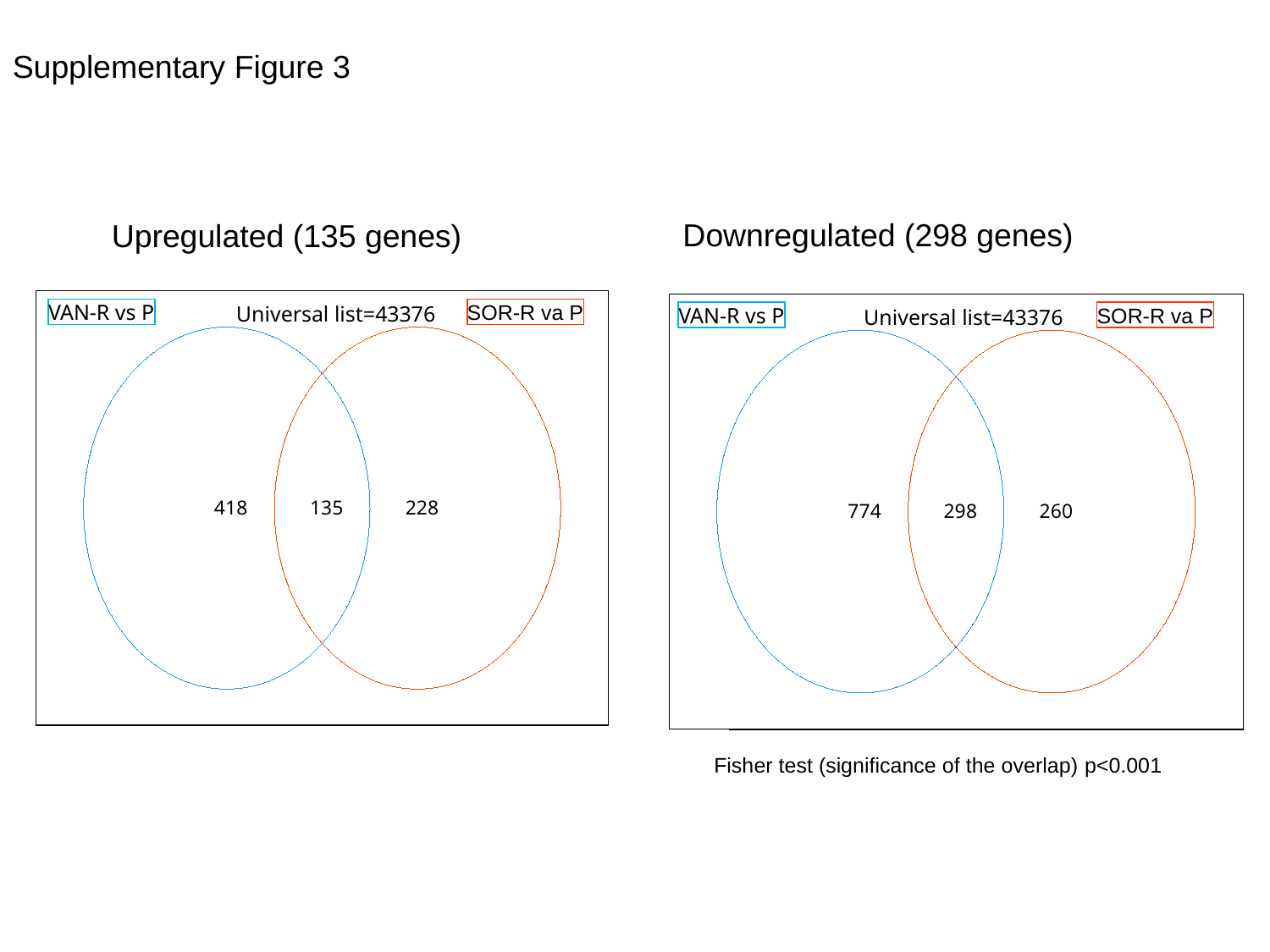

Supplementary Figure 3
Downregulated (298 genes)
Upregulated (135 genes)
VAN-R vs P
SOR-R va P
Universal list=43376
VAN-R vs P
SOR-R va P
Universal list=43376
418
135
228
774
298
260
Fisher test (significance of the overlap) p<0.001
