# Supplementary material for: Antitumour efficacy of MEK inhibitors in human lung cancer cells and their derivatives with acquired resistance to different tyrosine kinase inhibitors
Source: Br J Cancer. 2011 Jul 12;105(3):382–92. doi: 10.1038/bjc.2011.244 (PMC3172903; doi:10.1038/bjc.2011.244)
Supplement: Supplementary Figure 4 [file bjc2011244x4.ppt]

## Slide 1
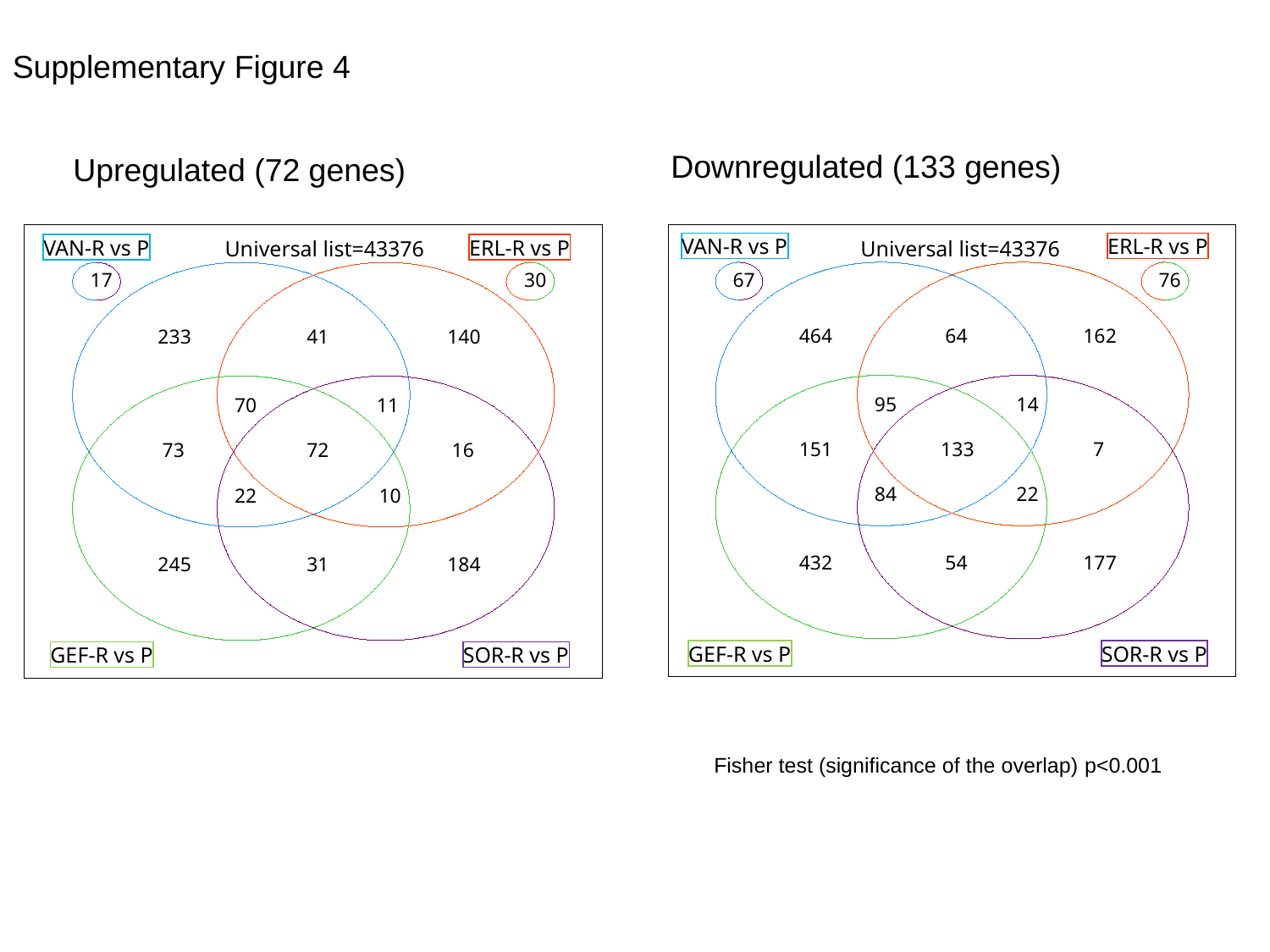

Supplementary Figure 4
Upregulated (72 genes)
Downregulated (133 genes)
VAN-R vs P
ERL-R vs P
VAN-R vs P
ERL-R vs P
Universal list=43376
Universal list=43376
67
76
17
30
464
64
162
233
41
140
95
14
70
11
151
133
7
73
72
16
84
22
22
10
432
54
177
245
31
184
GEF-R vs P
SOR-R vs P
GEF-R vs P
SOR-R vs P
Fisher test (significance of the overlap) p<0.001
