# Supplementary material for: Antitumour efficacy of MEK inhibitors in human lung cancer cells and their derivatives with acquired resistance to different tyrosine kinase inhibitors
Source: Br J Cancer. 2011 Jul 12;105(3):382–92. doi: 10.1038/bjc.2011.244 (PMC3172903; doi:10.1038/bjc.2011.244)
Supplement: Supplementary Figure 5 [file bjc2011244x5.ppt]

## Slide 1
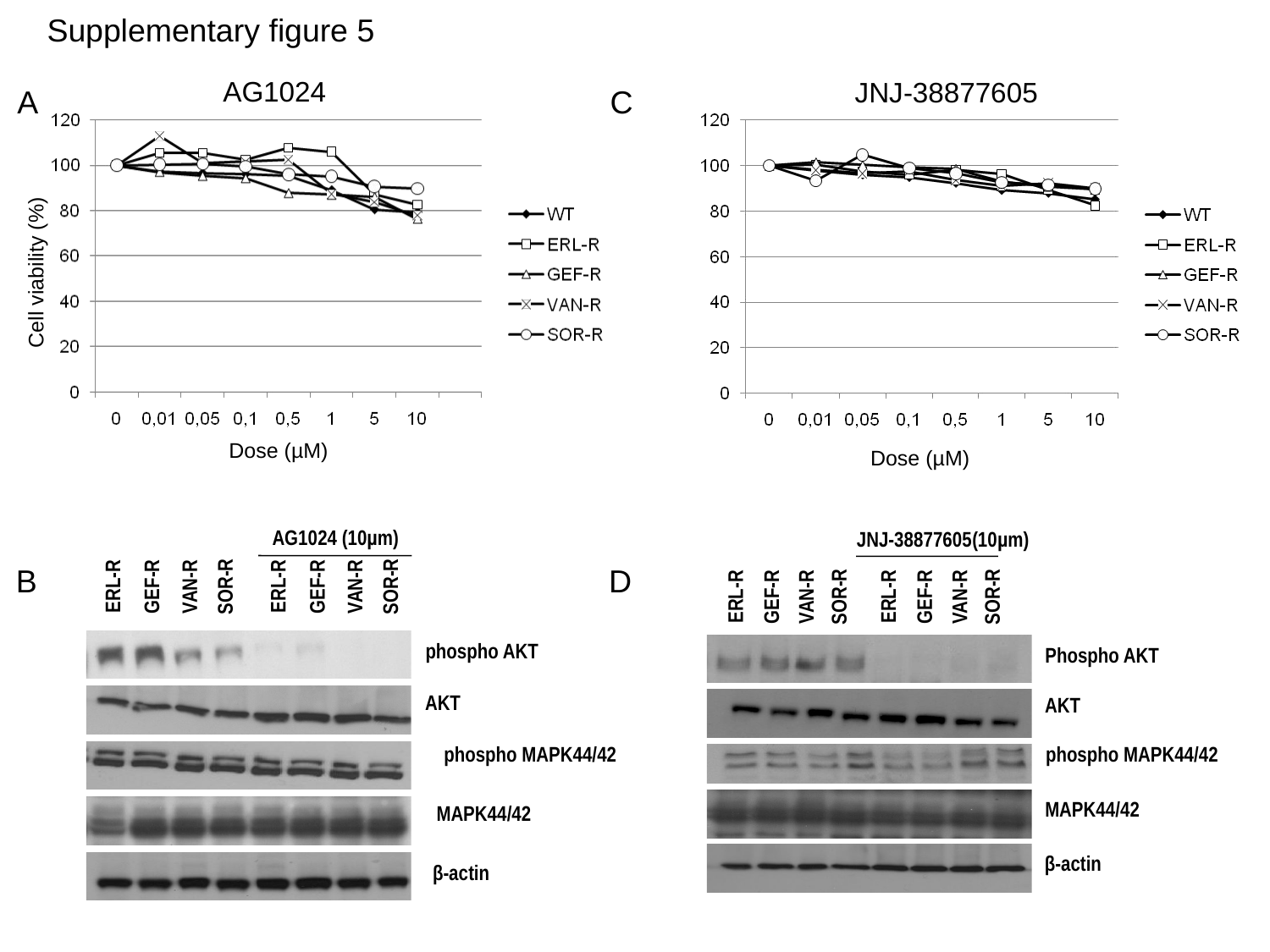

Supplementary figure 5
AG1024
Cell viability (%)
Dose (µM)
JNJ-38877605
Dose (µM)
A
C
AG1024 (10µm)
JNJ-38877605(10µm)
B
D
ERL-R
VAN-R
SOR-R
ERL-R
VAN-R
SOR-R
GEF-R
GEF-R
SOR-R
SOR-R
ERL-R
VAN-R
ERL-R
VAN-R
GEF-R
GEF-R
phospho AKT
Phospho AKT
AKT
AKT
phospho MAPK44/42
phospho MAPK44/42
MAPK44/42
MAPK44/42
β-actin
β-actin
