# Supplementary material for: Antitumour efficacy of MEK inhibitors in human lung cancer cells and their derivatives with acquired resistance to different tyrosine kinase inhibitors
Source: Br J Cancer. 2011 Jul 12;105(3):382–92. doi: 10.1038/bjc.2011.244 (PMC3172903; doi:10.1038/bjc.2011.244)
Supplement: Supplementary Table 1A [file bjc2011244x6.ppt]

## Slide 1
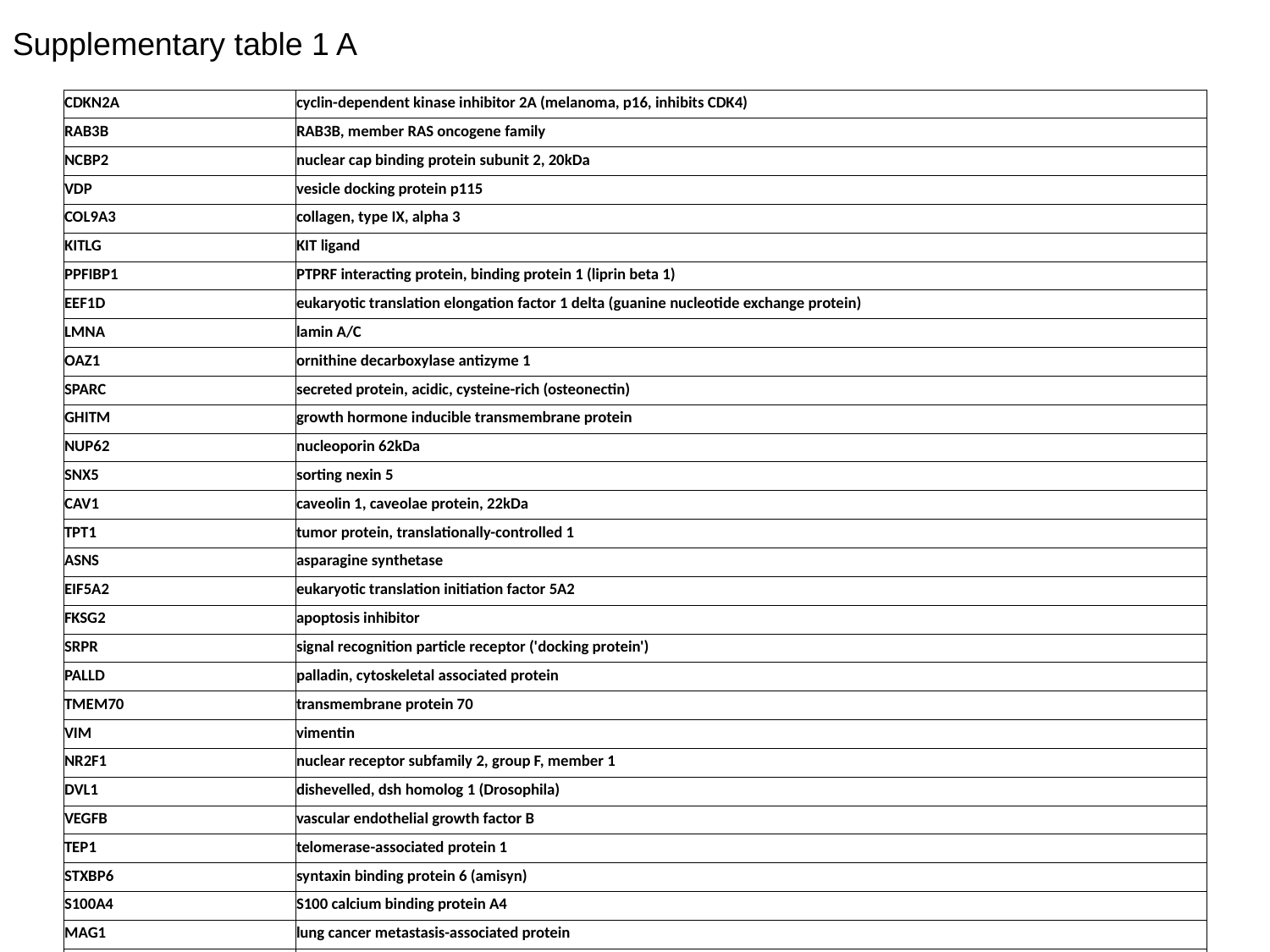

Supplementary table 1 A
| CDKN2A | cyclin-dependent kinase inhibitor 2A (melanoma, p16, inhibits CDK4) |
| --- | --- |
| RAB3B | RAB3B, member RAS oncogene family |
| NCBP2 | nuclear cap binding protein subunit 2, 20kDa |
| VDP | vesicle docking protein p115 |
| COL9A3 | collagen, type IX, alpha 3 |
| KITLG | KIT ligand |
| PPFIBP1 | PTPRF interacting protein, binding protein 1 (liprin beta 1) |
| EEF1D | eukaryotic translation elongation factor 1 delta (guanine nucleotide exchange protein) |
| LMNA | lamin A/C |
| OAZ1 | ornithine decarboxylase antizyme 1 |
| SPARC | secreted protein, acidic, cysteine-rich (osteonectin) |
| GHITM | growth hormone inducible transmembrane protein |
| NUP62 | nucleoporin 62kDa |
| SNX5 | sorting nexin 5 |
| CAV1 | caveolin 1, caveolae protein, 22kDa |
| TPT1 | tumor protein, translationally-controlled 1 |
| ASNS | asparagine synthetase |
| EIF5A2 | eukaryotic translation initiation factor 5A2 |
| FKSG2 | apoptosis inhibitor |
| SRPR | signal recognition particle receptor ('docking protein') |
| PALLD | palladin, cytoskeletal associated protein |
| TMEM70 | transmembrane protein 70 |
| VIM | vimentin |
| NR2F1 | nuclear receptor subfamily 2, group F, member 1 |
| DVL1 | dishevelled, dsh homolog 1 (Drosophila) |
| VEGFB | vascular endothelial growth factor B |
| TEP1 | telomerase-associated protein 1 |
| STXBP6 | syntaxin binding protein 6 (amisyn) |
| S100A4 | S100 calcium binding protein A4 |
| MAG1 | lung cancer metastasis-associated protein |
| NFKBIA | nuclear factor of kappa light polypeptide gene enhancer in B-cells inhibitor, alpha |
| TNFRSF6B | tumor necrosis factor receptor superfamily, member 6b, decoy |
| EEF1D | eukaryotic translation elongation factor 1 delta (guanine nucleotide exchange protein) |
| ADAM19 | ADAM metallopeptidase domain 19 (meltrin beta) |
| RAN | RAN, member RAS oncogene family |
| COL13A1 | collagen, type XIII, alpha 1 |
| HIF1A | hypoxia-inducible factor 1, alpha subunit (basic helix-loop-helix transcription factor) |
| RHOA | ras homolog gene family, member A |
