# Supplementary material for: Antitumour efficacy of MEK inhibitors in human lung cancer cells and their derivatives with acquired resistance to different tyrosine kinase inhibitors
Source: Br J Cancer. 2011 Jul 12;105(3):382–92. doi: 10.1038/bjc.2011.244 (PMC3172903; doi:10.1038/bjc.2011.244)
Supplement: Supplementary Table 1B [file bjc2011244x7.ppt]

## Slide 1
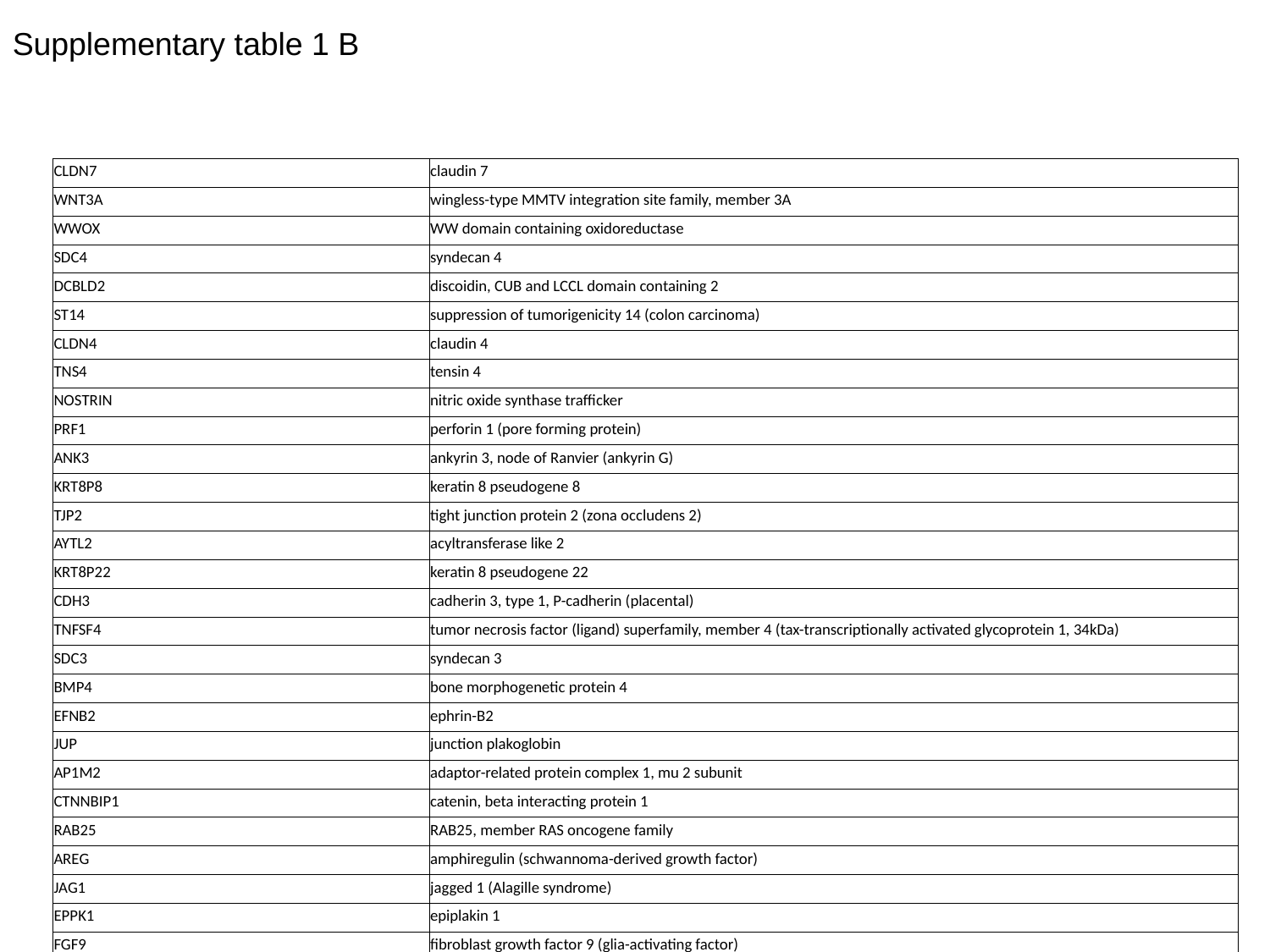

Supplementary table 1 B
| CLDN7 | claudin 7 |
| --- | --- |
| WNT3A | wingless-type MMTV integration site family, member 3A |
| WWOX | WW domain containing oxidoreductase |
| SDC4 | syndecan 4 |
| DCBLD2 | discoidin, CUB and LCCL domain containing 2 |
| ST14 | suppression of tumorigenicity 14 (colon carcinoma) |
| CLDN4 | claudin 4 |
| TNS4 | tensin 4 |
| NOSTRIN | nitric oxide synthase trafficker |
| PRF1 | perforin 1 (pore forming protein) |
| ANK3 | ankyrin 3, node of Ranvier (ankyrin G) |
| KRT8P8 | keratin 8 pseudogene 8 |
| TJP2 | tight junction protein 2 (zona occludens 2) |
| AYTL2 | acyltransferase like 2 |
| KRT8P22 | keratin 8 pseudogene 22 |
| CDH3 | cadherin 3, type 1, P-cadherin (placental) |
| TNFSF4 | tumor necrosis factor (ligand) superfamily, member 4 (tax-transcriptionally activated glycoprotein 1, 34kDa) |
| SDC3 | syndecan 3 |
| BMP4 | bone morphogenetic protein 4 |
| EFNB2 | ephrin-B2 |
| JUP | junction plakoglobin |
| AP1M2 | adaptor-related protein complex 1, mu 2 subunit |
| CTNNBIP1 | catenin, beta interacting protein 1 |
| RAB25 | RAB25, member RAS oncogene family |
| AREG | amphiregulin (schwannoma-derived growth factor) |
| JAG1 | jagged 1 (Alagille syndrome) |
| EPPK1 | epiplakin 1 |
| FGF9 | fibroblast growth factor 9 (glia-activating factor) |
| SMARCE1 | SWI/SNF related, matrix associated, actin dependent regulator of chromatin, subfamily e, member 1 |
