# Supplementary material for: Antitumour efficacy of MEK inhibitors in human lung cancer cells and their derivatives with acquired resistance to different tyrosine kinase inhibitors
Source: Br J Cancer. 2011 Jul 12;105(3):382–92. doi: 10.1038/bjc.2011.244 (PMC3172903; doi:10.1038/bjc.2011.244)
Supplement: Supplementary Table 2A [file bjc2011244x8.ppt]

## Slide 1
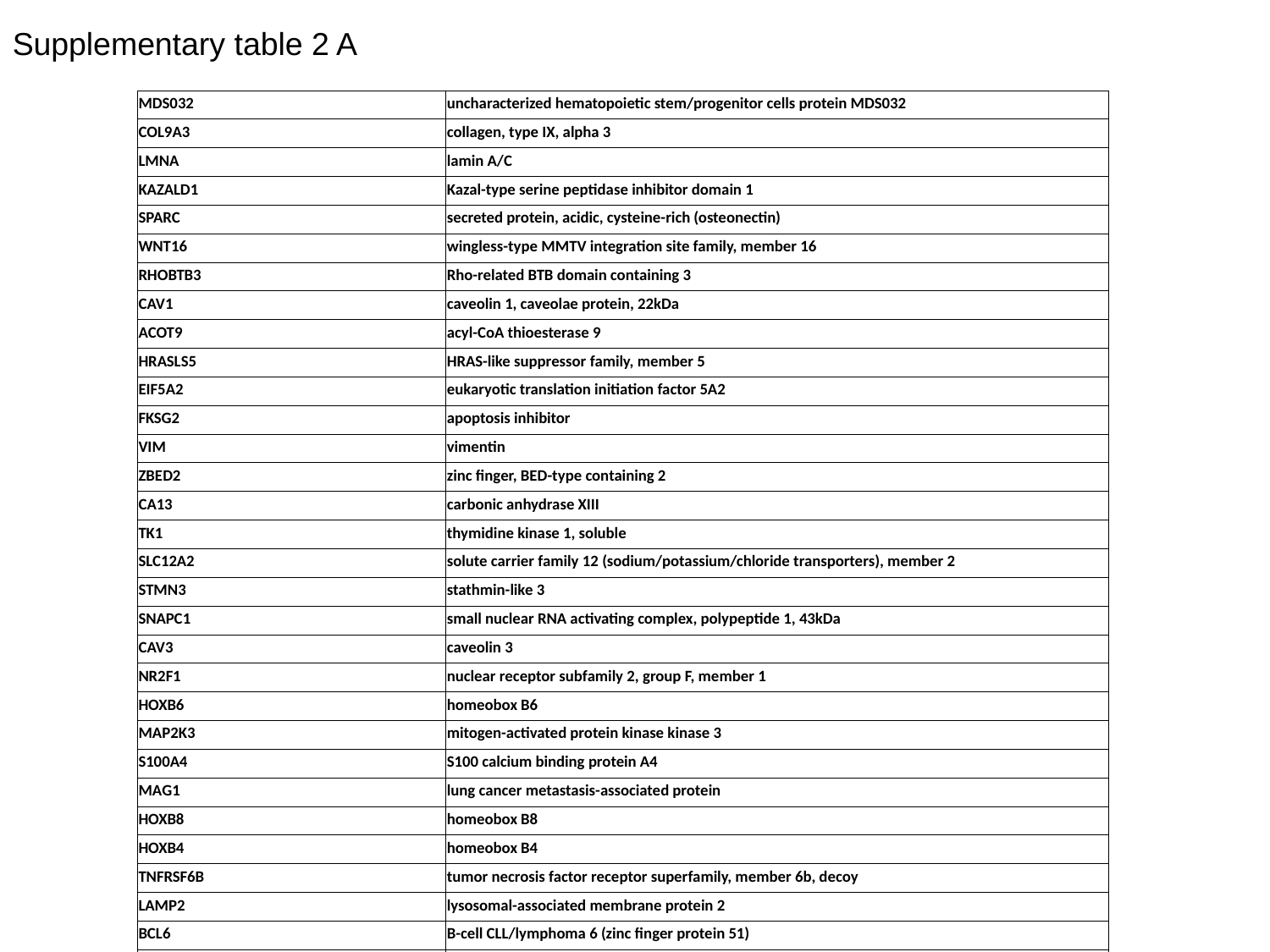

Supplementary table 2 A
| MDS032 | uncharacterized hematopoietic stem/progenitor cells protein MDS032 |
| --- | --- |
| COL9A3 | collagen, type IX, alpha 3 |
| LMNA | lamin A/C |
| KAZALD1 | Kazal-type serine peptidase inhibitor domain 1 |
| SPARC | secreted protein, acidic, cysteine-rich (osteonectin) |
| WNT16 | wingless-type MMTV integration site family, member 16 |
| RHOBTB3 | Rho-related BTB domain containing 3 |
| CAV1 | caveolin 1, caveolae protein, 22kDa |
| ACOT9 | acyl-CoA thioesterase 9 |
| HRASLS5 | HRAS-like suppressor family, member 5 |
| EIF5A2 | eukaryotic translation initiation factor 5A2 |
| FKSG2 | apoptosis inhibitor |
| VIM | vimentin |
| ZBED2 | zinc finger, BED-type containing 2 |
| CA13 | carbonic anhydrase XIII |
| TK1 | thymidine kinase 1, soluble |
| SLC12A2 | solute carrier family 12 (sodium/potassium/chloride transporters), member 2 |
| STMN3 | stathmin-like 3 |
| SNAPC1 | small nuclear RNA activating complex, polypeptide 1, 43kDa |
| CAV3 | caveolin 3 |
| NR2F1 | nuclear receptor subfamily 2, group F, member 1 |
| HOXB6 | homeobox B6 |
| MAP2K3 | mitogen-activated protein kinase kinase 3 |
| S100A4 | S100 calcium binding protein A4 |
| MAG1 | lung cancer metastasis-associated protein |
| HOXB8 | homeobox B8 |
| HOXB4 | homeobox B4 |
| TNFRSF6B | tumor necrosis factor receptor superfamily, member 6b, decoy |
| LAMP2 | lysosomal-associated membrane protein 2 |
| BCL6 | B-cell CLL/lymphoma 6 (zinc finger protein 51) |
| COL13A1 | collagen, type XIII, alpha 1 |
| CDK9 | cyclin-dependent kinase 9 (CDC2-related kinase) |
| HIF1A | hypoxia-inducible factor 1, alpha subunit (basic helix-loop-helix transcription factor) |
| EIF4H | eukaryotic translation initiation factor 4H |
| VCAN | versican |
| ADM | adrenomedullin |
| WDHD1 | WD repeat and HMG-box DNA binding protein 1 |
| AXL | AXL receptor tyrosine kinase |
| IL18 | interleukin 18 (interferon-gamma-inducing factor) |
