# Supplementary material for: Antitumour efficacy of MEK inhibitors in human lung cancer cells and their derivatives with acquired resistance to different tyrosine kinase inhibitors
Source: Br J Cancer. 2011 Jul 12;105(3):382–92. doi: 10.1038/bjc.2011.244 (PMC3172903; doi:10.1038/bjc.2011.244)
Supplement: Supplementary Table 2B [file bjc2011244x9.ppt]

## Slide 1
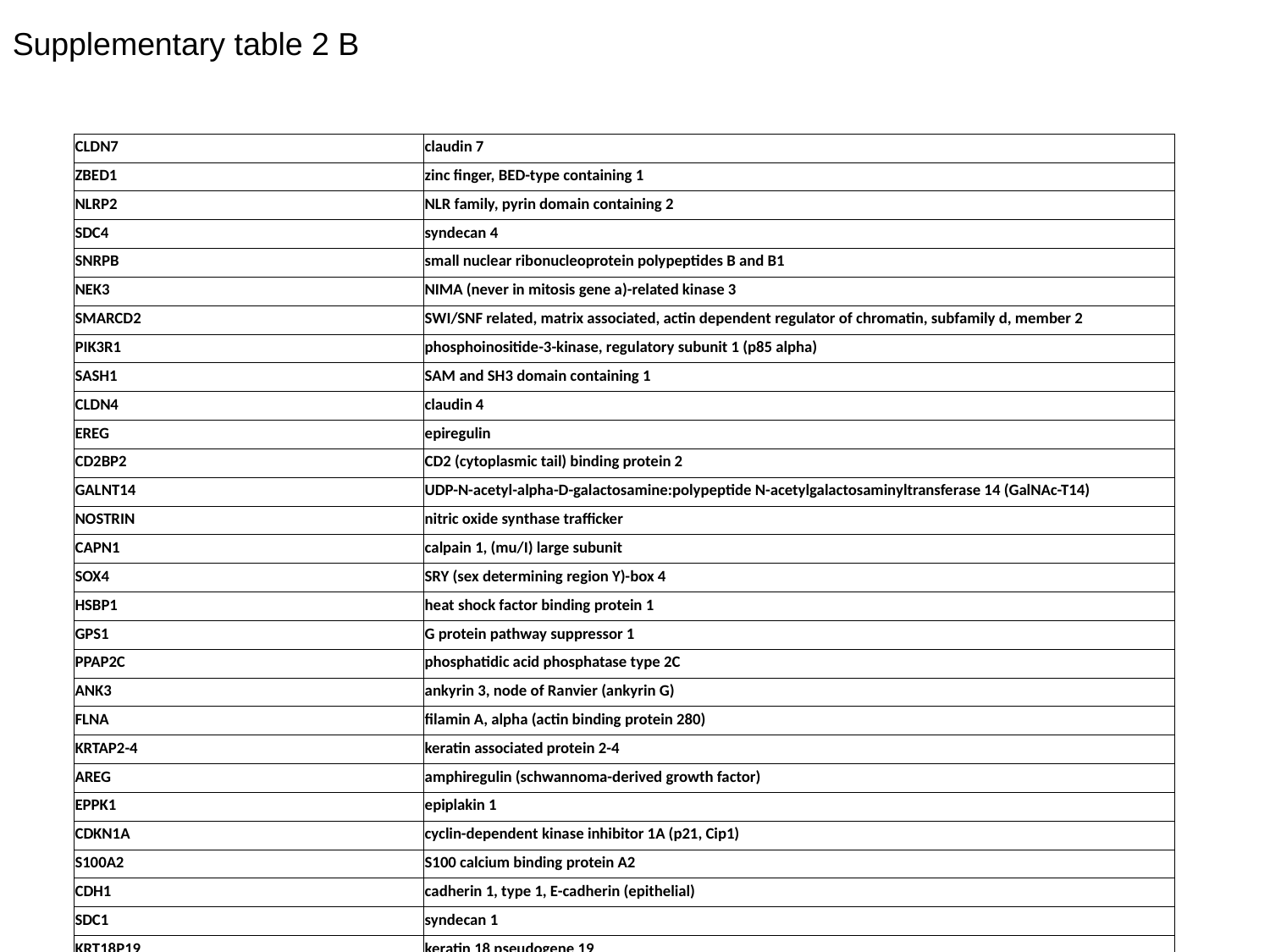

Supplementary table 2 B
| CLDN7 | claudin 7 |
| --- | --- |
| ZBED1 | zinc finger, BED-type containing 1 |
| NLRP2 | NLR family, pyrin domain containing 2 |
| SDC4 | syndecan 4 |
| SNRPB | small nuclear ribonucleoprotein polypeptides B and B1 |
| NEK3 | NIMA (never in mitosis gene a)-related kinase 3 |
| SMARCD2 | SWI/SNF related, matrix associated, actin dependent regulator of chromatin, subfamily d, member 2 |
| PIK3R1 | phosphoinositide-3-kinase, regulatory subunit 1 (p85 alpha) |
| SASH1 | SAM and SH3 domain containing 1 |
| CLDN4 | claudin 4 |
| EREG | epiregulin |
| CD2BP2 | CD2 (cytoplasmic tail) binding protein 2 |
| GALNT14 | UDP-N-acetyl-alpha-D-galactosamine:polypeptide N-acetylgalactosaminyltransferase 14 (GalNAc-T14) |
| NOSTRIN | nitric oxide synthase trafficker |
| CAPN1 | calpain 1, (mu/I) large subunit |
| SOX4 | SRY (sex determining region Y)-box 4 |
| HSBP1 | heat shock factor binding protein 1 |
| GPS1 | G protein pathway suppressor 1 |
| PPAP2C | phosphatidic acid phosphatase type 2C |
| ANK3 | ankyrin 3, node of Ranvier (ankyrin G) |
| FLNA | filamin A, alpha (actin binding protein 280) |
| KRTAP2-4 | keratin associated protein 2-4 |
| AREG | amphiregulin (schwannoma-derived growth factor) |
| EPPK1 | epiplakin 1 |
| CDKN1A | cyclin-dependent kinase inhibitor 1A (p21, Cip1) |
| S100A2 | S100 calcium binding protein A2 |
| CDH1 | cadherin 1, type 1, E-cadherin (epithelial) |
| SDC1 | syndecan 1 |
| KRT18P19 | keratin 18 pseudogene 19 |
| AKAP8L | A kinase (PRKA) anchor protein 8-like |
| MEGF8 | multiple EGF-like-domains 8 |
| PFKL | phosphofructokinase, liver |
| DCBLD2 | discoidin, CUB and LCCL domain containing 2 |
| PPARG | peroxisome proliferator-activated receptor gamma |
| NBL1 | neuroblastoma, suppression of tumorigenicity 1 |
| FGF9 | fibroblast growth factor 9 (glia-activating factor) |
