# Supplementary material for: Antitumour efficacy of MEK inhibitors in human lung cancer cells and their derivatives with acquired resistance to different tyrosine kinase inhibitors
Source: Br J Cancer. 2011 Jul 12;105(3):382–92. doi: 10.1038/bjc.2011.244 (PMC3172903; doi:10.1038/bjc.2011.244)
Supplement: Supplementary Table 3A [file bjc2011244x10.ppt]

## Slide 1
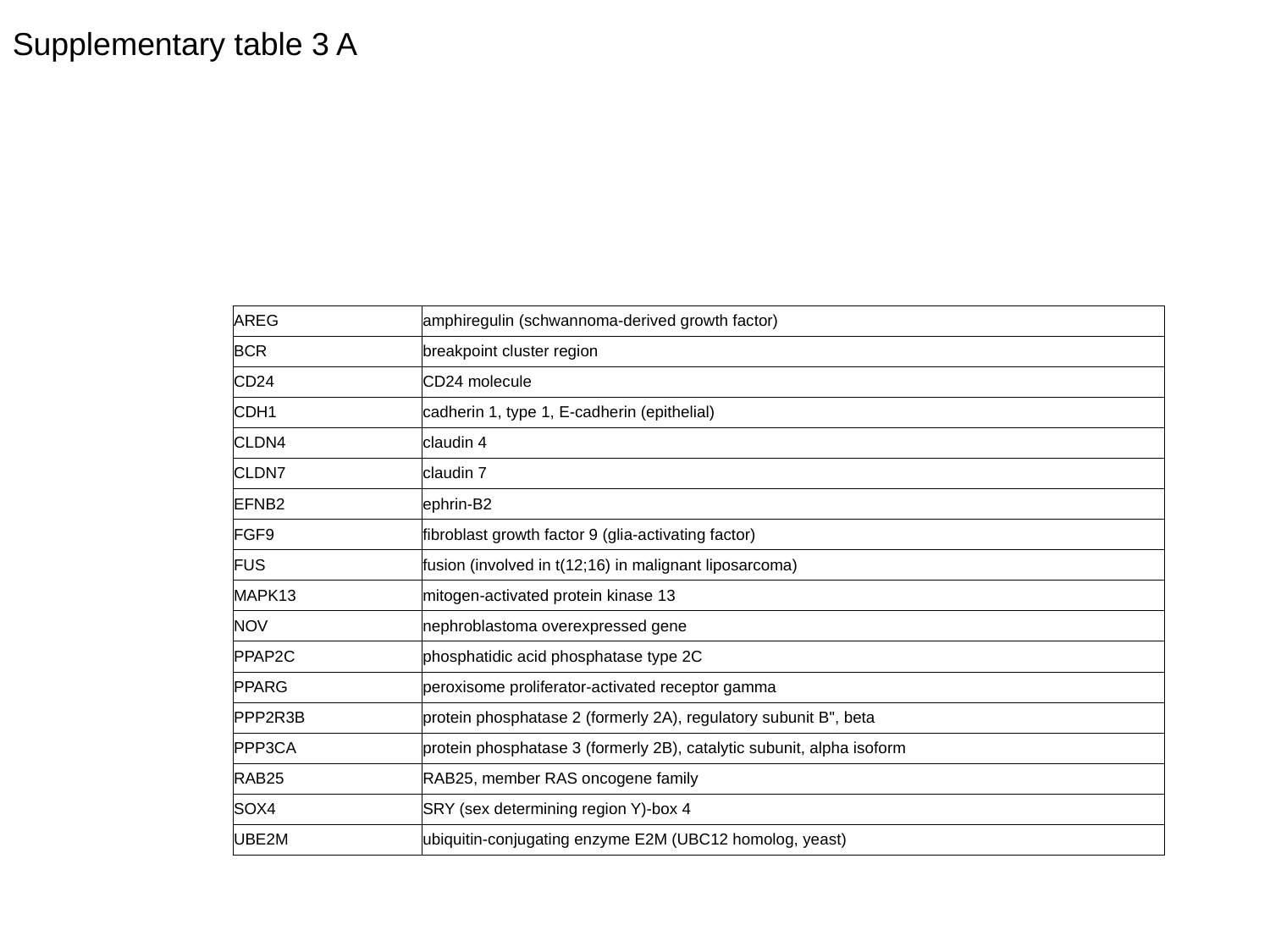

Supplementary table 3 A
| AREG | amphiregulin (schwannoma-derived growth factor) |
| --- | --- |
| BCR | breakpoint cluster region |
| CD24 | CD24 molecule |
| CDH1 | cadherin 1, type 1, E-cadherin (epithelial) |
| CLDN4 | claudin 4 |
| CLDN7 | claudin 7 |
| EFNB2 | ephrin-B2 |
| FGF9 | fibroblast growth factor 9 (glia-activating factor) |
| FUS | fusion (involved in t(12;16) in malignant liposarcoma) |
| MAPK13 | mitogen-activated protein kinase 13 |
| NOV | nephroblastoma overexpressed gene |
| PPAP2C | phosphatidic acid phosphatase type 2C |
| PPARG | peroxisome proliferator-activated receptor gamma |
| PPP2R3B | protein phosphatase 2 (formerly 2A), regulatory subunit B'', beta |
| PPP3CA | protein phosphatase 3 (formerly 2B), catalytic subunit, alpha isoform |
| RAB25 | RAB25, member RAS oncogene family |
| SOX4 | SRY (sex determining region Y)-box 4 |
| UBE2M | ubiquitin-conjugating enzyme E2M (UBC12 homolog, yeast) |
