# Supplementary material for: Antitumour efficacy of MEK inhibitors in human lung cancer cells and their derivatives with acquired resistance to different tyrosine kinase inhibitors
Source: Br J Cancer. 2011 Jul 12;105(3):382–92. doi: 10.1038/bjc.2011.244 (PMC3172903; doi:10.1038/bjc.2011.244)
Supplement: Supplementary Table 3B [file bjc2011244x11.ppt]

## Slide 1
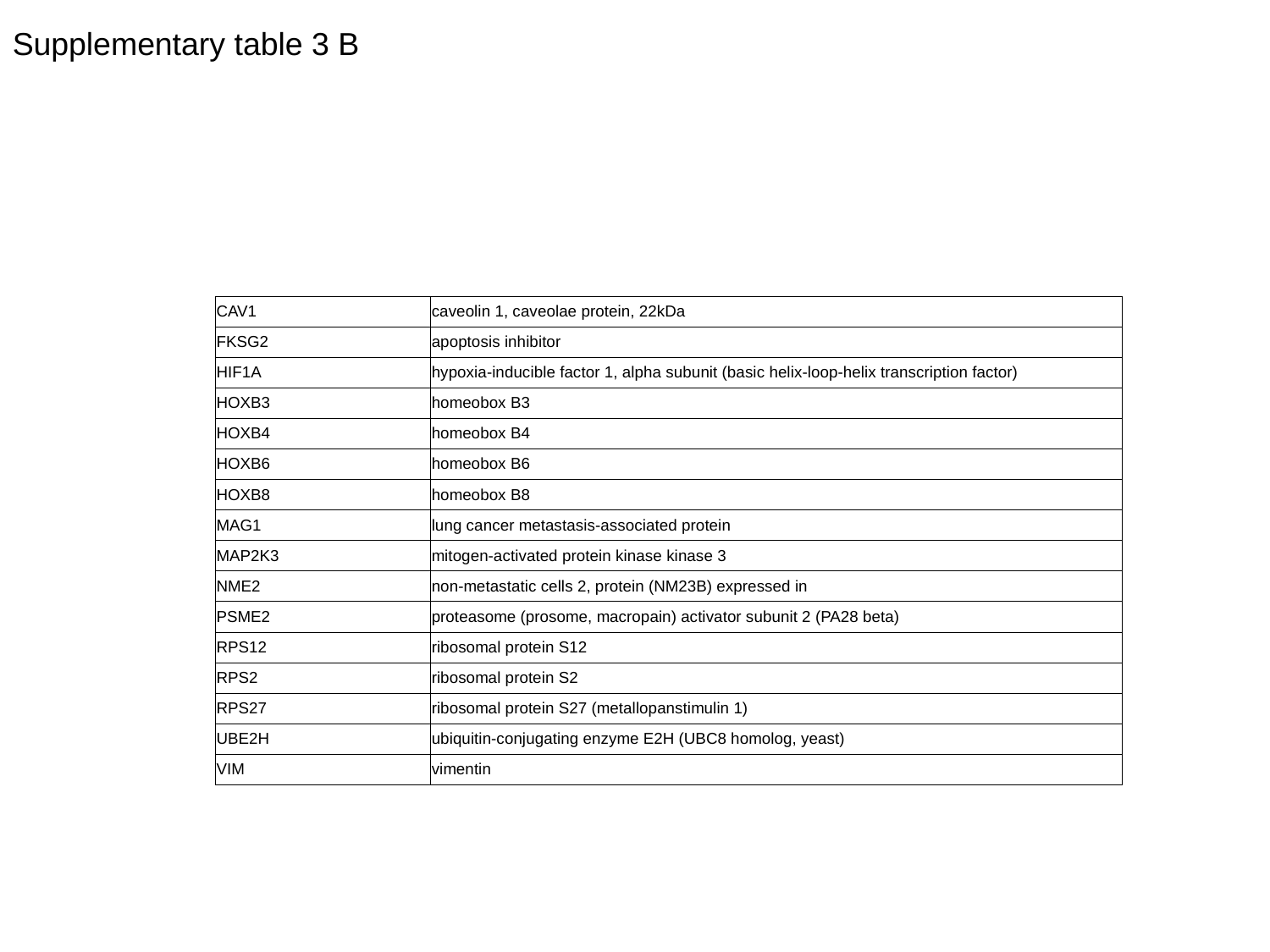

Supplementary table 3 B
| CAV1 | caveolin 1, caveolae protein, 22kDa |
| --- | --- |
| FKSG2 | apoptosis inhibitor |
| HIF1A | hypoxia-inducible factor 1, alpha subunit (basic helix-loop-helix transcription factor) |
| HOXB3 | homeobox B3 |
| HOXB4 | homeobox B4 |
| HOXB6 | homeobox B6 |
| HOXB8 | homeobox B8 |
| MAG1 | lung cancer metastasis-associated protein |
| MAP2K3 | mitogen-activated protein kinase kinase 3 |
| NME2 | non-metastatic cells 2, protein (NM23B) expressed in |
| PSME2 | proteasome (prosome, macropain) activator subunit 2 (PA28 beta) |
| RPS12 | ribosomal protein S12 |
| RPS2 | ribosomal protein S2 |
| RPS27 | ribosomal protein S27 (metallopanstimulin 1) |
| UBE2H | ubiquitin-conjugating enzyme E2H (UBC8 homolog, yeast) |
| VIM | vimentin |
