# Supplementary material for: Antitumour efficacy of MEK inhibitors in human lung cancer cells and their derivatives with acquired resistance to different tyrosine kinase inhibitors
Source: Br J Cancer. 2011 Jul 12;105(3):382–92. doi: 10.1038/bjc.2011.244 (PMC3172903; doi:10.1038/bjc.2011.244)
Supplement: Supplementary Information [file bjc2011244x12.doc]

**Supplementary Figure Legends**

**Supplementary Figure 1.**

**Expression of EGF-related growth factors and growth factor receptors in human CALU-3 lung adenocarcinoma cells.** Specific mRNA expression by quantitative Real-Time PCR (qRT-PCR): total RNA was extracted from CALU-3 NSCLC cell line and qRT PCR was done to assess the expression of amphiregulin, EGF, TGFα, EGFR, ERBB2, ERBB3, ERBB4, VEGF-A, VEGF-B, VEGF-C, VEGFR-1, VEGFR-2 and VEGFR-3 mRNAs. GEO colon cancer cells were used as a reference cell line (Martinelli et al 2010). Relative mRNA levels are expressed as compared to mRNA levels in GEO cancer cells.

**Supplementary Figure 2.**

**Identification of gene expression profiles in ERL-R and GEF-R CALU-3 human cancer cells.** For each drug-resistant cell line the log 2-fold-change in mRNA expression using Agilent microarrays (see Materials and Methods) between resistant ERL-R and GEF-R and sensitive P CALU-3 cell lines was evaluated. Probes with an absolute log 2-fold-change equal or superior to 0.5 were included in subsequent analyses. Venn diagrams were generated to study the overlap between genes and probes up- or down-regulated in cells resistant to erlotinib and gefitinib. **A** and **B,** Schematic representation of the most relevant up-regulated and down-regulated genes whose expression changes overlap between ERL-R and GEF-R CALU-3 cell lines as compared to P CALU-3 cells.

**Supplementary Figure 3.**

**Identification of gene expression profiles in VAN-R and SOR-R CALU-3 human cancer cells.** For each drug-resistant cell line the log 2-fold-change in mRNA expression using Agilent microarrays (see Materials and Methods) between resistant VAN-R and SOR-R and sensitive P CALU-3 cell lines was evaluated. Probes with an absolute log 2-fold-change equal or superior to 0.5 were included in subsequent analyses. Venn diagrams were generated to study the overlap between genes and probes up- or down-regulated in cells resistant to vandetanib and sorafenib. **A** and **B,** Schematic representation of the most relevant up-regulated and down-regulated genes whose expression changes overlap between VAN-R and SOR-R CALU-3 cell lines as compared to P CALU-3 cells.

**Supplementary Figure 4.**

**Identification of gene expression profiles in all four TKI-resistant CALU-3 human cancer cells.** As described above in the legend to Supplementary Figures 3 and 4, for each drug-resistant cell line the log 2-fold-change in mRNA expression using Agilent microarrays (see Materials and Methods) between resistantERL-R, GEF-R, VAN-R and SOR-R and sensitive P CALU-3 cell lines was evaluated. Probes with an absolute log 2-fold-change equal or superior to 0.5 were included in subsequent analyses. Venn diagrams were generated to study the overlap between genes and probes up- or down-regulated in cells resistant to erlotinib, gefitinib, vandetanib and sorafenib. **A** and **B,** Schematic representation of the most relevant up-regulated and down-regulated genes whose expression changes overlap between ERL-R, GEF-R, VAN-R and SOR-R CALU-3 cell lines as compared to P CALU-3 cells.

**Supplementary Figure 5.**

**Growth inhibitory effects of treatment with selective IGF1R and MET inhibitors in parental and TKI-resistant CALU-3 cancer cells.** MTT cell proliferation assays were performed in parental lung adenocarcinoma CALU-3 cells (P) and in their TKI-resistant derivatives (ERL-R, GEF-R, VAN-R and SOR-R), treated for three days with the indicated concentrations of each of two selective IGF1R (**A**) and MET inhibitors (**C**). Western blotting analysis of AKT and MAPK activation following treatment with the indicated concentration of each of two selective inhibitors, AG1024 (**B**) or JNJ 38877605 (**D**) on TKI-resistant CALU-3. β-actin was included as a loading control.

**Supplementary Tables**

**Supplementary Table 1.**

**A**, Selected list of genes whose mRNA expression is up-regulated in both ERL-R and GEF-R CALU-3 cell lines as compared to WT CALU-3 cells. **B**, Selected list of genes whose mRNA expression is down-regulated in both ERL-R and GEF-R CALU-3 cell lines as compared to WT CALU-3 cells.

**Supplementary Table 2.**

**A**, Selected list of genes whose mRNA expression is up-regulated in both VAN-R and SOR-R CALU-3 cell lines as compared to WT CALU-3 cells. **B**, Selected list of genes whose mRNA expression is down-regulated in both VAN-R and SOR-R CALU-3 cell lines as compared to WT CALU-3 cells.

**Supplementary Table 3.**

**A**, Selected list of genes whose mRNA expression is up-regulated in all four resistant (ERL-R, GEF-R, VAN-R, SOR-R) CALU-3 cell lines as compared to WT CALU-3 cells. **B**, Selected list of genes whose mRNA expression is down-regulated in all four resistant (ERL-R, GEF-R, VAN-R, SOR-R) CALU-3 cell lines as compared to WT CALU-3 cells.
